# Ribotype Classification of *Clostridioides difficile* Isolates Is Not Predictive of the Amino Acid Sequence Diversity of the Toxin Virulence Factors TcdA and TcdB

**DOI:** 10.3389/fmicb.2020.01310

**Published:** 2020-06-19

**Authors:** Zhenghui Li, Kwok Lee, Urvi Rajyaguru, C. Hal Jones, Sandra Janezic, Maja Rupnik, Annaliesa S. Anderson, Paul Liberator

**Affiliations:** ^1^Vaccine Research and Development, Pfizer Inc., Pearl River, NY, United States; ^2^National Laboratory for Health, Environment and Food, Maribor, Slovenia; ^3^Faculty of Medicine, University of Maribor, Maribor, Slovenia

**Keywords:** *Clostridioides difficile*, *Clostridium difficile*, TcdA, TCDB, vaccines, whole genome sequencing

## Abstract

*Clostridioides* (*Clostridium*) *difficile* is the most commonly recognized cause of infectious diarrhea in healthcare settings. Currently there is no vaccine to prevent initial or recurrent *C. difficile* infection (CDI). Two large clostridial toxins, TcdA and TcdB, are the primary virulence factors for CDI. Immunological approaches to prevent CDI include antibody-mediated neutralization of the cytotoxicity of these toxins. An understanding of the sequence diversity of the two toxins expressed by disease causing isolates is critical for the interpretation of the immune response to the toxins. In this study, we determined the whole genome sequence (WGS) of 478 *C. difficile* isolates collected in 12 countries between 2004 and 2018 to probe toxin variant diversity. A total of 44 unique TcdA variants and 37 unique TcdB variants were identified. The amino acid sequence conservation among the TcdA variants (≥98%) is considerably greater than among the TcdB variants (as low as 86.1%), suggesting that different selection pressures may have contributed to the evolution of the two toxins. Phylogenomic analysis of the WGS data demonstrate that isolates grouped together based on ribotype or MLST code for multiple different toxin variants. These findings illustrate the importance of determining not only the ribotype but also the toxin sequence when evaluating strain coverage using vaccine strategies that target these virulence factors. We recommend that toxin variant type and sequence type (ST), be used together with ribotype data to provide a more comprehensive strain classification scheme for *C. difficile* surveillance during vaccine development objectives.

## Introduction

*Clostridioides* (*Clostridium*) *difficile*, a Gram-positive, spore-forming, obligate anaerobe, is the main cause of nosocomial infectious diarrhea in industrialized countries (Kelly et al., 1994). The bacterium accounts for 20–30% of cases of antibiotic-associated diarrhea and is the most commonly recognized cause of infectious diarrhea in healthcare settings (Cohen et al., 2010). The main risk factors for an initial episode of *C. difficile* infection (CDI) are antibiotic therapy, hospitalization, and underlying comorbidities. Older adults (≥65 years of age) are at increased risk for CDI, particularly when exposed to health care settings (Asempa and Nicolau, 2017).

*C. difficile* can produce 3 toxins, toxin A (TcdA), toxin B (TcdB), and binary toxin (CDT) (Kuehne et al., 2014). TcdA and TcdB are large single subunit proteins (approximately 308 and 270 kDa, respectively), and are considered the principal virulence factors contributing to CDI (Voth and Ballard, 2005). These two toxins have similar structural features delineated by four functional domains but share just 50% overall amino acid sequence identity. The C-terminal domain of the toxin proteins, known as the combined repetitive oligopeptide (CROP) domain, facilitates toxin binding to the surface of intestinal epithelial cells. The toxins enter the cell by endocytosis where the reduced pH of endocytic vesicles triggers a conformational change in the cell entry domain of the toxin, resulting in pore formation and translocation of the glucosyltransferase domain (GTD) and autoprocessing domain (APD) to the cytosolic face of the membrane. Binding of the cytosolic cofactor InsP6 activates the APD, resulting in cleavage and release of the GTD. GTD-catalyzed transfer of glucose inactivates small cytoplasmic GTPases of the Rho family of proteins, leading to the disruption of the cytoskeleton. This manifests as a cytopathic rounding effect and cell death in the epithelium which results in diarrhea. CDT belongs to the family of binary ADP-ribosylating toxins consisting of two components: CDTa (ADP-ribosyltransferase) and CDTb (responsible for host cell binding and translocation of CDTa to the cytosol). As *cdtA* and *cdtB* are not detected in all toxigenic isolates, the significance of CDT as a virulence factor contributing to CDI remains in question (Gerding et al., 2014).

*C. difficile* infection often occurs when the integrity of the normal intestinal microbiota is disturbed. The spectrum of CDI presentation includes mild self-limiting to severe diarrhea which may progress to pseudomembranous colitis, toxic megacolon, intestinal perforation, and death (Bartlett, 2002). Although most patients experiencing a first episode of CDI respond well to standard antibiotic treatment (which can include metronidazole, vancomycin or fidaxomicin), approximately 15–35% of patients suffer from at least one recurrence (Louie et al., 2011). Immunoprophylactic approaches that target *C. difficile* toxins have been developed for the prevention of recurrent CDI (Sheldon et al., 2016). Vaccines have been successfully developed to prevent other toxin mediated diseases, such as tetanus and diphtheria, by inducing antibodies that neutralize the cytopathic effect of the toxin (Looney et al., 1956). The proposed mechanism of action of these approaches is through antibody mediated (both monoclonal antibody as well as vaccine-elicited polyclonal responses) neutralization of the cytotoxic activity of toxins produced by disease-causing *C. difficile* isolates.

Several molecular methods have been used to type *C. difficile* isolates for epidemiological studies. These include restriction endonuclease analysis (REA), pulse field gel electrophoresis (PFGE), and ribotyping, a PCR-based method that takes advantage of the size heterogeneity of the intergenic spacer region (ISR) between 16S and 23S rRNA genes (Loo et al., 2005; McDonald et al., 2005; Goorhuis et al., 2008). Isolates can also be classified by toxinotype, a restriction fragment length polymorphism (RFLP) method that is based on changes in the *C. difficile* pathogenicity locus (PaLoc) (Rupnik and Janezic, 2016). *C. difficile* may also be grouped into five main clades and at least three additional cryptic clades based on the clustering of the concatenate multiple locus sequence typing (MLST) alleles (Janezic et al., 2018). While each of these methods provides value for isolate characterization in epidemiological studies, none provides detailed insight to the diversity of full length TcdA and TcdB proteins. Reports on sequence-based variability within TcdA and TcdB toxins in large strain collections are uncommon. Sequence diversity within a 199 amino acid fragment of the CROP domain of TcdB (corresponding to the receptor binding domain of the toxin), has been used to differentiate toxin variant types (Dingle et al., 2011). An understanding of toxin diversity among contemporary disease-causing isolates is essential for assessment of the immune response to the toxins. In this manuscript we report on the deduced amino acid sequence of TcdA and TcdB toxin proteins determined from whole genome nucleotide sequence data of 478 *C. difficile* isolates. A total of 44 TcdA and 37 TcdB protein variants have been identified among these isolates and are presented together with detailed molecular analyses.

## Materials and Methods

### Strain Collection, Isolate Selection, Microbiology and DNA Isolation

Initially, a total of 504 *C. difficile* isolates, collected from multiple sources across several geographic regions between the years 2004–2018 were included in this study (Table 1). The isolates do not represent a prevalence-based collection. Whole genome sequence (WGS) data was collected for all isolates and the PCR ribotype was determined for each of the isolates. Toxinotype assignments were available for a subset of the isolates.

**TABLE 1:** *Clostridioides difficile* isolates included in this study.

Collection name	Collection year	Geographic origin	Molecular typing	Number of isolates
Legacy North America	2004–2009	United States, Canada	Ribotype, WGS	24
Legacy United Kingdom	<2011	United Kingdom	Ribotype, WGS	26
Antimicrobial Testing Leadership and Surveillance (ATLAS)	2009–2017	Belgium, Czech Republic, France, Germany, Hungary, Spain, Sweden	Ribotype, WGS	303
Toxinotype diversity subset	2009–2016	Australia, Belgium, Germany, Slovenia, Spain, United Kingdom	Ribotype, Toxinotype, WGS	64
United States contemporary	2015–2018	United States	Ribotype, WGS	87

*C. difficile* isolates were either provided as glycerol stocks or were purified from stool specimens. All microbiology was conducted under anaerobic conditions. Glycerol stocks were plated onto Tryptone Soya Agar (TSA) + 5% sheep blood and incubated overnight at 37°C. On day 2, a single colony was selected and re-streaked onto a new TSA +5% sheep blood agar plate and again incubated overnight at 37°C. On day 3, colonies were transferred to a 96-well plate for lysis and genomic DNA was extracted using Beckman Coulter GenFind V2 kit (Indianapolis, IN, United States). For stool specimens, a 10 μl loop was used to transfer stool to 700 μL 95% ethanol; 20 μL of this suspension was then used to inoculate a cycloserine cefoxitin fructose agar plate, supplemented with horse blood and taurocholate (CCFA-HT) and incubated for 2 days at 37°C. On day 3, a single colony was picked from CCFA-HT plate and streaked onto a TSA blood agar plate followed by overnight incubation at 37°C. On day 4, the colonies were transferred to a 96-well plate for lysis and genomic DNA was extracted using the Beckman Coulter GenFind V2 kit.

### Molecular Characterization of the Study Isolates

For the majority of strains, PCR-ribotyping was performed using the protocol described in Svenungsson et al. (2003). For added discrimination, PCR products are analyzed using an Agilent 2100 Bioanalyzer. Ribotype and toxinotype assignments for the toxiontype diversity subset of strains were determined as described in Bidet et al. (1999) and Rupnik et al. (1998), respectively. The nucleotide sequence of *adk, atpA, dxr, glyA, recA, sodA*, *and tpi* genes was extracted from the whole genome sequence of each strain and used to assign a sequence type (ST) and clade at PubMLST^1^.

### Preparation of the Illumina Sequencing Library

*C. difficile* sequencing libraries were prepared by using either the TruSeq DNA Sample Prep Kits (“TruSeq”) or the Nextera DNA Flex Library Prep Kit (“Flex”), both from Illumina (San Diego, CA, United States). When using the TruSeq kit, genomic DNAs are first mechanically sheared by a Covaris ME220 (Woburn, MA, United States) sonication instrument using the settings recommended by the manufacturer. After sonication, the TruSeq universal adapters are added to the ends of the genomic DNA fragments by ligation, followed by bead cleanup, size selection and quantification according to the manufacturer’s protocol. When using the Flex kit, genomic DNAs are first tagmented (transposon-mediated fragmentation) and universal adapters are added to the ends of the DNA fragments by PCR, according to the manufacturer’s protocol. DNA libraries (paired end, 2 × 300) were loaded on the Illumina Miseq for whole genome sequencing of the respective *C. difficile* genomes.

### Analysis of the WGS Data

Primary nucleotide sequence data for the 478 *C. difficile* isolates from this study has been deposited to NCBI SRA (BioProject PRJNA600974). Primary *C. difficile* DNA sequence reads were run through the “Merge Overlapping Pairs” program followed by assembly into contigs using the “De Novo Assembly” program in the Qiagen CLC Genomics Workbench (Redwood City, CA, United States) using default parameters. The *tcdA* and *tcdB* allele sequences were inferred by aligning the assembled contigs to the *tcdA* and *tcdB* sequences of the CD630 isolate (GenBank accession AM180355) (Sebaihia et al., 2006). The deduced amino acid sequences of the genes coding for TcdA and TcdB in CD630 are labeled as TcdA001 and TcdB001, respectively.

A unique toxin variant is defined as a TcdA or TcdB open reading frame (ORF) that includes all four of the functional domains and whose amino acid sequence differs by at least one amino acid from any other entry. The most challenging region for collection of high-quality DNA sequence was in the C-terminal CROP domain, particularly in the TcdA ORF. In some instances, these challenges were addressed by repeat sequencing with fewer input strains to achieve greater depth and/or by performing the *de novo* assembly program a second time without merging overlapping pairs. If the toxin variant could not be determined by the *de novo* assembly program, primary sequence data was aligned to the CD630 reference genome to confirm the presence of the gene.

The nomenclature developed by den Dunnen et al. (2016) has been used to describe a series of truncations and in-frame deletions predicted from the *tcdA* nucleotide sequence of some isolates. In these instances, the TcdA001 sequence is used as the reference when describing position in the ORF. A unique TcdA variant number has been assigned if sequence corresponding to the CROP domain is included in the truncated ORF. However, if the deduced amino acid sequence is truncated prior to the CROP domain, a TcdA variant was not assigned and the isolate has been labeled as “truncated at TcdA.” In those isolates where the entire *tcdA* sequence is missing, the strain has been classified as “TcdA deletion.” Acceptance criteria for each novel TcdA or TcdB variant identified required a minimum 35X nucleotide sequence coverage across the respective ORFs with less than 10% sequence heterogeneity. If these acceptance criteria were not met, the isolate was not included in the phylogenomic analysis. The WGS from 26 of the 504 isolates in the collection did not meet these acceptance criteria and were not included in these analyses. TcdA and TcdB amino acid sequences were aligned using CLC Genomics Workbench and aligned sequences were used to construct the phylogenetic tree using the unweighted pair group method with arithmetic mean (UPGMA) with bootstrapping. The phylogenomic figure was created by aligning assembled *C. difficile* genomes using Parsnp (Treangen et al., 2014) with recombination filtration option enabled. The metadata (clade, ST, ribotype, TcdA variant type, and TcdB variant type) was plotted as concentric rings using a custom-built R program.

### Measurement of Toxin Variant Diversity and Distribution Within Phylogenetic Clades

The Simpson’s Diversity Index (SDI) was used to quantitatively measure the diversity and distribution of toxin variants within phylogenetic clades of *C. difficile*. The higher the index obtained, the greater the number and distribution of the toxin variants observed within a clade. Likewise, the lower the index, the fewer number of variants and/or a more restricted distribution of the toxin variants within a clade.

## Results

### Phylogenetic Analysis of TcdA and TcdB Variants

**FIGURE 1:**
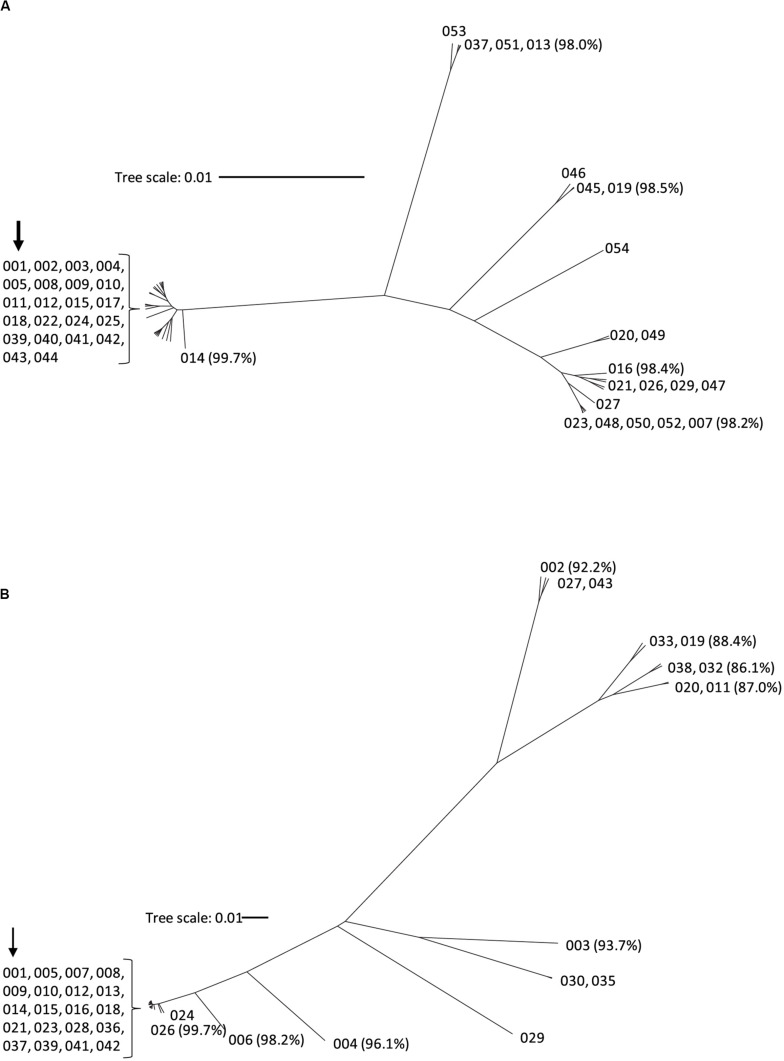
Phylogenetic tree of TcdA **(A)** and TcdB **(B)** toxin variants. The amino acid sequences of the 44 TcdA variants and 37 TcdB variants were used to build the phylogenetic trees using CLC Genomics workbench with the UPGMA method. Each branch represents a unique toxin variant. The length of each branch corresponds to the amino acid sequence diversity from the TcdA001 **(A)** or TcdB001 **(B)** variants. The numbers in parentheses represent the pairwise amino acid sequence identity of representative variants to TcdA001 or TcdB001. The position of the TcdA001 and TcdB001 variant sequences from the CD630 isolate is highlighted with an arrow.

Nucleotide sequence corresponding to *tcdA* and *tcdB* was extracted from the WGS data and used to generate the deduced amino acid sequence of TcdA and TcdB protein variants, respectively. To serve as a point of reference, the TcdA and TcdB proteins coded for by strain CD630 are variants TcdA001 and TcdB001. A total of 43 unique TcdA variants that differ from variant TcdA001 were identified. All TcdA variants were closely related, with pairwise amino acid sequence identity to TcdA001 that ranged from 98.0 to >99.9%. From the deduced amino acid sequence of TcdA variants, an unrooted phylogenetic tree was constructed using the UPGMA (Figure 1A). The toxin variant used as template to make the TxdA vaccine antigen (TcdA001) is grouped together with 21 other TcdA variants, with each variant sharing >99.7% amino acid sequence identity with TcdA001. Of the TcdA variants whose ORF is at least 2,710 amino acids in length, TcdA013 was the most diverse (98.0% sequence identity with TcdA001) and is grouped with three others. Variant TcdA007 and TcdA016 (98.2 and 98.4% sequence identity with TcdA001, respectively), are phylogenetically related to several other full-length variants. Finally, TcdA019 which shares 98.5% identity with TcdA001, is representative of a fourth group of variants on the TcdA phylogenetic tree. Most of the sequence variation among the 44 TcdA variants is due to single amino acid substitutions. The greatest amino acid sequence diversity among the TcdA variants is found within the CROP domain of the proteins (Table 2). The *tcdA* nucleotide sequence of 25 isolates predict ORFs that are shorter than TcdA001 (summarized in Supplementary Table 1). A termination codon in the TcdA sequence from 14 isolates occurs following amino acid residue 46 (p.Q47^∗^) (von Eichel-Streiber et al., 1999). While these isolates were collected in different geographic regions, including the United States and multiple European countries, each is genotyped as ST37/RT017. The *tcdA* sequence of three additional isolates also predicted termination codons within the GTD domain of the toxin (p.V57^∗^, p.D108^∗^, p.P196^∗^). A common termination codon (p.G699^∗^) in the APD domain is shared by three isolates. Since the predicted TcdA ORFs of these 20 isolates lack at least 3 of the functional toxin domains, TcdA variants were not assigned. Novel TcdA variants were assigned for 5 isolates whose *tcdA* sequence predict a deletion of a portion of the CROP domain of the toxin. In-frame deletions of 33 (TcdA050) and 175 amino acids (TcdA051) were detected in two isolates, while three isolates had termination codons in the TcdA CROP domain (TcdA052, TcdA053, TcdA054). Critical catalytic residues within the GTD (D285 and D287) and APD domains (C700) of the toxins are conserved among each of the 44 unique TcdA variants.

**TABLE 2:** Pairwise amino acid sequence identity (%) of a subset of diverse TcdA and TcdB variants to TcdA001 and TcdB001, respectively.

	Entire protein	Glucosyl transferase	Auto protease	Cell entry	Binding (CROP)
**TcdA variant**					
TcdA013	98.0	99.5	99.2	98.6	96.1
TcdA007	98.2	99.5	99.6	98.6	96.5
TcdA020	98.2	99.5	99.6	98.8	96.3
TcdA019	98.5	99.6	99.2	99.0	96.8
TcdA014	99.7	100	100	99.6	99.7
**TcdB variant**					
TcdB032	86.1	79.2	90.2	87.9	87.6
TcdB011	87.0	79.4	90.2	89.4	88.4
TcdB019	88.4	79.2	90.6	91.7	90.4
TcdB002	92.2	96.5	97.3	90.6	88.4
TcdB003	93.7	79.0	90.6	99.2	99.4
TcdB015	99.8	100	100	99.9	99.4

There were 31 non-toxigenic isolates, lacking any sequence corresponding to TcdA and TcdB (TcdA−/TcdB−). These were not restricted to a single ST or RT. A subset of 3 strains code for a full length TcdB variant, but without any *tcdA* sequence (TcdA−/TcdB+). These TcdA−/TcdB+ strains were also associated with multiple ribotypes. Precedent for both TcdA−/TcdB+ as well as non-toxigenic TcdA−/TcdB− *C. difficile* strains is well established in the literature (Rupnik, 2008).

The amino acid sequences of TcdB variants were more diverse than TcdA. Of the 36 variants that differed from TcdB001, pairwise amino acid sequence identity with TcdB001 ranged from 86.1 to >99.9%. This is illustrated in the phylogenetic tree of TcdB variants (Figure 1B). A total of 19 variants group together with TcdB001 (>99.8% pairwise sequence identity). Variants TcdB032 and TcdB038 are the most diverse, sharing 86.1 and 86.2% sequence identity with TcdB001, respectively. TcdB032 is detected in one isolate in the collection (typed as ST62 and RT591) and two isolates code for TcdB038 (ST567 and RT095). While sequence diversity is detected in each of the functional domains of the TcdB toxin, the greatest diversity is found within the GTD domain (Table 2). Unlike TcdB001, eleven of the TcdB variants have a lysine inserted in the GTD domain (p.Val307_Thr308insLys). Based on the structure of the TcdB GTD domain (PDB ID 2BVM^2^) (Reinert et al., 2005), this additional amino acid residue is not predicted to impact the conformation of the GTD domain. The eleven sequence variants that contain the additional amino acid cluster to two groups on the TcdB phylogenetic tree (Figure 1B). Critical catalytic residues of GTD (D286 and D288) and autoprotease (C698) domains are conserved among all TcdB variants. Although the TcdA and TcdB toxins share similar architectural homology with respect to the functional domains of the molecules, the pairwise amino acid sequence identity between TcdA001 and TcdB001 is only 42%.

More than half of the isolates (*n* = 292, 61%) in this study did not code for binary toxin (Supplementary Table 2). Each of the 31 non-toxigenic TcdA−/TcdB− isolates was binary toxin negative (genotyped as *cdtA*−*/cdtB*−). Considerable differences in TcdA and TcdB variant type and prevalence were also noted when comparing binary toxin positive and negative subsets of isolates. With very few exceptions, TcdA and TcdB variant types were selectively associated with either binary positive or binary negative isolates. Exceptions include variants TcdA007 and TcdB002. TcdA007 is coded for by just one of 292 binary negative isolates (0.3%) and 94/186 (50.5%) binary positive isolates. Similarly, the gene coding for TcdB002 is detected in just one binary negative strain but in 108/186 (58.1%) binary positive strains. This is particularly noteworthy as the *C. difficile* pathogenicity locus is not linked to the binary toxin alleles (Gerding et al., 2014).

### Phylogenomic Analysis and the Association of *C. difficile* Epidemiological Markers With Toxin Variant Type

**FIGURE 2:**
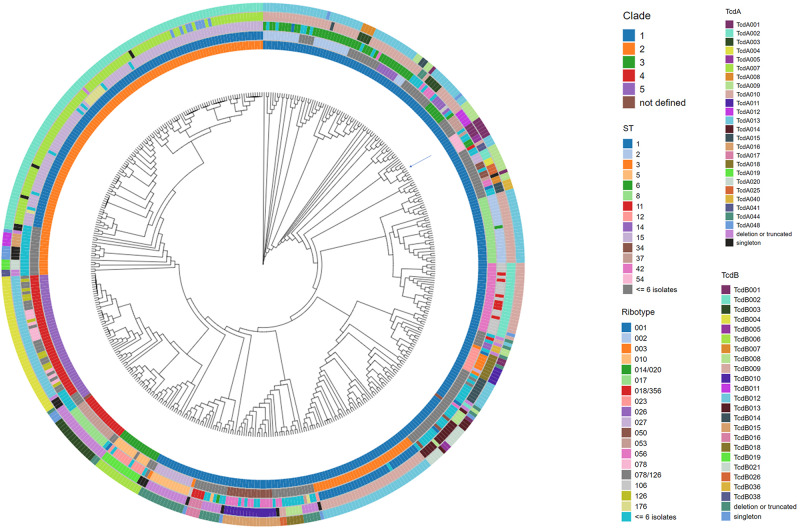
Phylogenomic tree of *C. difficile* isolates with accompanying meta data. The whole genome sequence of *C. difficile* isolates was used to construct a tree to illustrate the phylogenomic relationship among the isolates. *C. difficile* isolate CD630 (GenBank accession AM180355) was used as the reference genome during the alignment. The position of the CD630 genome is highlighted by an arrow on the tree. Each branch of the dendrogram tree corresponds to a *C. difficile* isolate in the collection. From the innermost circle to the outermost circle, the five color-coded concentric circles identify clade, ST, ribotype, TcdA, and TcdB variant type assignments for each isolate. For clarity, ST and ribotype assignments identified in less than or equal to 6 isolates were colored as dark gray and light blue, respectively. Clade assignment could not be made in two instances (entered as not defined). TcdA and TcdB variants detected just once in the collection (singletons) are colored as black and blue, respectively.

The 478 *C. difficile* genomes were assembled to construct a phylogenomic tree using Parsnp (Figure 2). The nucleotide sequence of isolate CD630 was used as the reference genome. Each branch of the dendrogram is representative of a *C. difficile* isolate. Metadata descriptive of each isolate (Supplementary Table 2), including clade, ST, ribotype and toxin variant type, has been added as concentric rings to the circumference of the phylogenomic tree. Included among the 478 isolates are 61 ribotypes and 71 sequence types (STs). Comparative analysis afforded by the figure helps to illustrate that these epidemiological markers are not predictive of toxin variant type. Starting at 12 o’clock on the tree and traveling counterclockwise to 10 o’clock is a cluster of 107 isolates that are typed as ST1 and all code for TcdB variant TcdB002. Although most isolates in this cluster are ribotype 027, other ribotypes such as 081, 176, 027/198, and 198 are also identified. Continuing counter-clockwise on the tree is a cluster of 42 isolates that are typed as ST11 and code for TcdB variant TcdB004. Despite sharing considerable phylogenomic, ST and TcdB variant type similarities, the ribotype variability among these isolates is considerable, including ribotypes 045, 078, 126, 078/126, and 413.

Among the numerous methods that have been developed for molecular typing of *C. difficile* isolates, ribotype is most commonly cited. Analysis of WGS data in this study illustrates that isolates grouped by ribotype code for sequence-diverse toxin variants (Tables 3, 4). Among the 21 isolates that are grouped as ribotype 078/126, genes coding for three TcdA variant types (TcdA013, TcdA015, TcdA046) were identified. The ribotype 078/126 isolates also code for three TcdB variants (TcdB004, TcdB006, TcdB012) whose amino acid sequence identity to TcdB001 ranges from 96.1 to 99.9%. Ribotype 014/020 isolates in the collection code for four unique TcdA and six TcdB variants. The pairwise identity of the six TcdB variants to TcdB001 ranges from 92.2 to 99.9%.

**TABLE 3:** *C. difficile* isolates grouped by ribotype can code for multiple and sequence diverse TcdA variants.

TcdA variant^1^	Ribotype (number of isolates)														

	001 (40)	002 (21)	003 (7)	013 (6)	014/020 (47)	018/356 (13)	027 (96)	053 (8)	056 (21)	070 (5)	078 (11)	078/126 (21)	106 (18)	126 (10)	258 (2)
001	–	–	–	–	–	–	–	1	–	–	–	–	–	–	–
002	–	–	–	–	–	7	3	–	–	–	–	–	16	–	–
003	–	–	–	–	3	–	–	–	4	–	–	–	–	–	–
005	–	–	–	–	2	–	–	–	–	–	–	–	–	–	–
007	–	–	–	–	–	–	81	–	–	–	–	–	–	–	–
008	–	–	–	–	–	–	–	–	2	–	–	–	–	–	–
009	–	–	–	1	–	–	–	–	–	–	–	–	–	–	–
010	35	21	–	1	40	2	–	–	2	–	–	–	1	–	1
011	–	–	–	–	–	–	–	–	11	–	–	–	–	–	1
012	–	–	–	–	–	–	–	5	–	–	–	–	–	–	–
013	–	–	–	–	–	–	–	–	–	–	11	18	–	10	–
014	1	–	–	3	–	–	–	–	1	–	–	–	–	–	–
015	1	–	–	–	1	–	–	–	–	5	–	2	–	–	–
017	–	–	–	–	–	4	–	–	–	–	–	–	–	–	–
018	1	–	6	–	–	–	1	–	–	–	–	–	–	–	–
022	–	–	1	–	–	–	–	–	–	–	–	–	–	–	–
023	–	–	–	–	–	–	1	–	–	–	–	–	–	–	–
024	–	–	–	–	–	–	–	–	–	–	–	–	1	–	–
025	–	–	–	1	–	–	–	–	–	–	–	–	–	–	–
039	–	–	–	–	–	–	–	1	–	–	–	–	–	–	–
042	–	–	–	–	–	–	–	–	1	–	–	–	–	–	–
046	–	–	–	–	–	–	–	–	–	–	–	1	–	–	–
048	–	–	–	–	–	–	8	–	–	–	–	–	–	–	–
050	–	–	–	–	–	–	1	–	–	–	–	–	–	–	–
052	–	–	–	–	–	–	1	–	–	–	–	–	–	–	–
NC^2^	2	–	–	–	1	–	–	1	–	–	–	–	–	–	–

*^1^The number of isolates that code for the respective TcdA variants within each ribotype subset are enumerated. ^2^Isolates that do not code for full length TcdA proteins.*

**TABLE 4:** *C. difficile* isolates grouped by ribotype can code for multiple and sequence diverse TcdB variants.

TcdB Variant^1^	Ribotype (number of isolates)														

	001 (40)	002 (21)	003 (7)	013 (6)	014/020 (47)	018/356 (13)	027 (96)	053 (8)	056 (21)	070 (5)	078 (11)	078/126 (21)	106 (18)	126 (10)	258 (2)
001	1	–	1	–	2	–	–	2	–	–	–	–	–	–	–
002	–	–	–	–	–	–	92	–	–	–	–	–	–	–	–
004	–	–	–	–	–	–	–	–	–	–	11	18	–	10	–
006	–	–	–	–	–	–	–	–	–	–	–	1	–	–	–
007	–	–	–	–	3	–	–	–	–	–	–	–	–	–	–
008	–	–	–	1	3	–	3	5	2	–	–	–	–	–	–
009	–	–	–	–	–	7	–	–	–	–	–	–	15	–	–
010	–	–	4	–	–	–	1	–	–	–	–	–	–	–	–
012	35	21	–	1	36	2	–	–	5	5	–	2	–	–	–
013	–	–	–	1	–	–	–	–	–	–	–	–	–	–	–
014	–	–	–	–	1	–	–	–	–	–	–	–	–	–	–
015	–	–	–	–	–	–	–	–	12	–	–	–	–	–	1
016	–	–	–	–	–	4	–	–	–	–	–	–	–	–	–
021	1	–	–	3	–	–	–	–	–	–	–	–	–	–	–
023	–	–	–	–	–	–	–	–	–	–	–	–	1	–	–
024	–	–	–	–	–	–	–	–	–	–	–	–	1	–	–
026	–	–	–	–	–	–	–	–	1	–	–	–	–	–	1
028	–	–	–	–	1	–	–	–	–	–	–	–	–	–	–
036	1	–	–	–	–	–	–	–	–	–	–	–	–	–	–
037	–	–	–	–	–	–	–	–	1	–	–	–	–	–	–
042	–	–	–	–	–	–	–	–	–	–	–	–	1	–	–
NC^2^	2	–	2	–	1	–	–	1	–	–	–	–	–	–	–

*^1^The number of isolates that code for the respective TcdB variants within each ribotype subset are enumerated. ^2^Isolates that do not code for full length TcdB proteins.*

Using ST assignments, all but two of the isolates from this study could be grouped into one of five clades. With the exception of clade 4, the number and distribution of TcdA variants within each of the clades is more diverse than TcdB variants (Supplementary Figure 1).

## Discussion

*C. difficile* infection is a worldwide public health issue and is now the most common healthcare-associated bacterial pathogen. It was estimated that during 2011 there were nearly half a million cases of CDI in the United States, associated with approximately 29,000 deaths (Lessa et al., 2015). Several vaccine strategies for the prevention of CDI have been, or are currently, being evaluated in clinical trials and most of these have focused on the large single subunit glucosylating toxins, TcdA and TcdB as antigens (Bezay et al., 2016). Clinical proof of concept for the selection of these toxins as *C. difficile* vaccine antigens has come from studies that were used to support the approval of Bezlotoxumab (ZINPLAVA^TM^, Merck) to reduce the recurrence of CDI in adult patients (Alonso and Mahoney, 2019). Bezlotoxumab is a human monoclonal antibody that binds to a discontinuous epitope located within the CROP domain of TcdB (Orth et al., 2014). Since the CROP domain accounts for approximately 20% of the entire protein, a prophylactic vaccine approach able to generate a functional polyclonal response to additional and multiple epitopes across the entire toxin has the potential to prevent primary disease. Three prophylactic vaccines composed of full-length toxin open reading frames, or portions thereof, have been evaluated in clinical studies. A recombinant fusion protein that includes a portion of the TcdA and TcdB CROP domain from a single strain of *C. difficile* (Bezay et al., 2016) has completed phase 2 testing (NCT02316470). The antigens included in the other investigational vaccines correspond to inactivated full length toxin proteins (i.e., toxoids TxdA, and TxdB) (Donald et al., 2013). Following a planned interim analysis in a phase 3 clinical study for one of the toxoid based vaccines (NCT01887912), the study was discontinued based on the low probability of meeting its primary objective (Sanofi, 2017). A bivalent toxoid-based vaccine composed of genetically and chemically inactivated toxoid antigens is in phase 3 clinical development (NCT03918629). Active immunization with these genetically and chemically modified full length versions of TcdA and TcdB has elicited a robust polyclonal antibody response in subjects 65–85 years of age (Sheldon et al., 2016).

Several molecular methods including WGS have been utilized to characterize *C. difficile* toxin genes for epidemiological analysis of CDI (Huber et al., 2013; Knetsch et al., 2013; Janezic and Rupnik, 2019). Two of these, toxinotype (Rupnik and Janezic, 2016) and the deduced amino acid sequence of the Receptor Binding Domain (RBD) of TcdB variants (Dingle et al., 2011), have focused on toxin genotype of *C. difficile* isolates. However, neither method provides detailed insight into the sequence diversity of the complete open reading frame of the two large toxin proteins. An understanding of the toxin variants expressed by CDI isolates is critically important for an assessment of the breadth of the functional antibody response elicited following immunization with a bivalent toxoid vaccine. Demonstration that the polyclonal immune sera can neutralize the cytotoxicity of sequence diverse toxins is required for an evaluation of vaccine efficacy. Prediction of the breadth of the functional immune response to a toxin-based vaccine antigen needs to be framed in the context of toxin variant type determined from WGS, because isolate classification by ribotype is not representative of the diversity of toxin protein sequences.

The availability of *C. difficile* WGS data with deep sequence coverage, across the PaLoc in particular, is limited. In a recent study, analysis of WGS data from 906 *C. difficile* strains focused on questions related to bacterial adaptation for healthcare-mediated transmission, but did not investigate the diversity of the *tcdA* and *tcdB* alleles (Kumar et al., 2019). The WGS of 478 *C. difficile* isolates have been determined in our study. Acceptance criteria for toxin variant assignment required greater than 35X coverage across both *tcdA* and *tcdB* with less than 10% sequence heterogeneity at any nucleotide position for at least one representative isolate. A total of 44 unique TcdA variants and 37 unique TcdB variants were identified, many of which had not previously been reported. The toxin variants with pairwise amino acid sequence identity furthest removed from TcdA001 and TcdB001 were TcdA013 (98% sequence identity) and TcdB032 (86.1% sequence identity), respectively. The ability of the immune response elicited by a toxoid antigen to neutralize the cytotoxicity of sequence diverse toxins remains to be experimentally tested. It should be noted that the origin of the *C. difficile* isolates evaluated in this study was largely restricted to North America and Europe. The TcdA and TcdB sequences of isolates from different geographic regions may uncover further toxin diversity. In addition, as the isolates collected in this study are not prevalence based the most frequent TcdA and TcdB variant types identified here may not be representative of prevalence among circulating CDI isolates.

Although both TcdA and TcdB play a role in CDI, several observations suggest that TcdB is the major virulence factor. In both murine and hamster models of *C. difficile* infection, TcdB was responsible for most of the intestinal damage whereas TcdA caused more superficial and localized damage (Carter et al., 2015). It is also worth noting that TcdA+/TcdB− *C. difficile* strains are extremely rare (Monot et al., 2015), whereas TcdA−/TcdB+ strains are not uncommon and have been associated with multiple disease outbreaks (Drudy et al., 2007; Rupnik, 2008). The greater amino acid sequence diversity noted among the 37 TcdB variants compared with the 44 TcdA variants identified in this study could in part be the consequence of host immune pressure applied to the more virulent TcdB toxins.

Several isolates with deduced amino acid sequences shorter than TcdA001 have been identified in this collection. The majority of these are the product of in-frame stop codons in the glucosyl transferase or auto protease domains and therefore are not likely to code for a functional toxin. TcdA variants with either a deletion or a termination codon within the CROP domain have also been identified. Cell-culture based cytotoxicity assays are required to demonstrate whether these TcdA variants are functional. Previous work by Rupnik and Janezic has indicated that toxinotype VI and VII strains, with deletions in the CROP domain, do produce functional TcdA (Rupnik and Janezic, 2016). It has also been reported that a TcdA mutant lacking the entire CROP domain is cytotoxic, able to enter human cells and cause cytotoxicity, albeit with reduced uptake (Gerhard et al., 2013). Among the isolates evaluated in this study, similar genetic changes in the sequences corresponding to the CROP domain of TcdB variants have not been identified. The larger number and length of repeat units in the TcdA CROP domain may render this locus more prone to genetic rearrangements than TcdB. Differences between the genes coding for the two toxins extend beyond the CROP domain. Examples of toxigenic isolates lacking any sequence coding for TcdA (TcdA−/TcdB+) are included in this collection and have been described in the literature (von Eichel-Streiber et al., 1999). As noted earlier and despite their close proximity in the PaLoc, examples of TcdA+/TcdB− isolates, lacking *tcdB* sequences are not common (Monot et al., 2015). Our current understanding of the molecular detail of epithelial cell binding and uptake also differentiates the toxins from one another (Chandrasekaran and Lacy, 2017). Taken together, these observations suggest that evolution of the two toxins has been and will continue to be subject to different selection pressures. Relative to the highly conserved TcdA variants, the greater sequence diversity among TcdB variants described in this manuscript is consistent with this idea.

The same acceptance criteria for deep sequence coverage were applied in the analysis of WGS data corresponding to the genes coding for both subunits of the *C. difficile* binary toxin. The majority of isolates (*n* = 292, 61%) in this study did not code for binary toxin (e.g., genotyped as *cdtA*−*/cdtB*−). Consistent with literature reports indicating that CDI isolates typed as TcdA−/TcdB−/CDT+ are rare (Geric et al., 2003; Gerding et al., 2014), there were no TcdA−/TcdB−/CDT+ isolates identified in this study. A study in a hamster model of CDI suggested that vaccination with bivalent TxdA and TxdB antigens did not fully protect from challenge with a TcdA+/TcdB+/CDT+ NAP1 strain and that the addition of binary toxin antigens to the vaccine greatly enhanced protection (Secore et al., 2017). Interestingly, human epidemic isolates 027/BI/NAP1 and 078/BK/NAP7 are both positive for binary toxin (Gerding et al., 2014), whereas the emerging RT106 as well as RT017 isolates common in Asia are negative for binary toxin (Liu et al., 2018; Imwattana et al., 2019). The contribution of binary toxin to clinical CDI remains controversial.

*C. difficile* isolates can be grouped into five different clades based on ST assignments (Griffiths et al., 2010). Dingle et al. showed that 17 alleles coding for 13 deduced peptide sequences corresponding to the TcdB RBD segregate in parallel with clade assignment (Dingle et al., 2011). That is, no single RBD allele was found in more than one clade. However, this study focused on just a small section of TcdB and did not capture the full sequence diversity of TcdB variants. In addition, the diversity of TcdA has not been taken into consideration. Using ST assignments from WGS data collected in our study, 476 of the 478 isolates can be placed into one of the 5 clades. Greater than half of the isolates (*n* = 276) are grouped into Clade 1 and these are genotypically diverse, including 49 different STs, 39 ribotypes, 24 TcdA variants and 22 TcdB variants. However, much like the clade-restricted observation for the RBD alleles, none of the 24 TcdA and 22 TcdB variants are coded for by strains other than those grouped to clade 1. The same conclusion can be drawn for strains associated with each clade. For example, while clade 5 strains are fewer in number (*n* = 45) and much less diverse (42 of these are ST11 and each code for TcdB004), all TcdA013 and TcdB004 variants are coded for by strains that are grouped to clade 5.

The Simpson’s Diversity Index (SDI) analysis indicated that the distribution of TcdA variants within the respective clades is generally more diverse than the variants of TcdB (Supplementary Figure 1). Although both TcdA and TcdB are co-localized to the PaLoc, the variation in SDI suggested that TcdA and TcdB might have evolved in response to different evolutionary pressures. One interpretation is that point mutations in TcdA are sporadic and equally represented across phylogenetic clades (excluding clade 4 in this collection of strains). The variability noted among TcdB protein sequences can be postulated as the sum of sporadic point mutations together with pressure exerted from additional mechanisms. It is interesting to note that while three distinct cell surface receptors for TcdB have been identified, [PVRL3 (or NECTIN3), CSPG4 and members of the Frizzled protein family] (LaFrance et al., 2015; Yuan et al., 2015; Tao et al., 2016), similar molecular binding detail has not been uncovered for TcdA. In fact, none of these receptors bind TcdA. In contrast to the CROP domain of TcdA, high-affinity glycan mediated binding for TcdB CROP at the cell surface has not been experimentally demonstrated (Gerhard, 2017). The molecular difference in host cell entry mechanisms utilized by TcdA and TcdB may contribute to the variation of SDI noted here.

To our knowledge, this is the first comprehensive WGS characterization of a large *C. difficile* isolate collection with a focused analysis of the sequence diversity among TcdA and TcdB toxins. From WGS data, we identified 44 TcdA variants and 37 TcdB variants, many of which were not previously documented. The relative lack of deep WGS data deposited in public databases could be due to technical challenges associated with the sequencing of *C. difficile* isolates using conventional methods, and in particular sequence determination across repetitive regions of the pathogenicity locus. We observed that neither ribotype nor ST assignment is predictive of the toxin genotype of *C. difficile* isolates. Specifically, isolates that are grouped together based on ribotype assignment can code for multiple sequence diverse TcdA and TcdB variants. We propose an amended nomenclature that describes both the ribotype and the TcdA and TcdB variant type for the purpose of *C. difficile* strain surveillance during vaccine efficacy trials. As the immune response to toxoid antigens will be polyclonal and not restricted to the RBD, it is essential that sequence diversity of the entire toxin proteins be considered in efforts to estimate vaccine efficacy. While challenges are associated with obtaining deep sequence coverage across the pathogenicity locus, WGS should be applied for *C. difficile* isolate typing together with ribotype determination to better guide *C. difficile* epidemiological studies and to help evaluate immunological approaches for the prevention of *C. difficile* disease.

## Data Availability Statement

The datasets generated for this study can be found in the NCBI BioProject ID PRJNA600974.

## Author Contributions

ZL and PL contributed substantially to conception and design and acquisition of the data, conducted the analysis and interpretation of the data, and drafted and revised the manuscript, and gave final approval of the version to be published, and agreed to act as guarantor of the work (ensuring that questions related to any part of the work are appropriately investigated and resolved). AA, MR, and SJ contributed substantially to conception and design, analysis and interpretation of the data, and revised the article critically for important intellectual content and gave final approval of the version to be published, and agreed to act as guarantor of the work (ensuring that questions related to any part of the work are appropriately investigated and resolved). KL, UR, and CJ contributed substantially to acquisition of the data and revised the manuscript critically for important intellectual content and gave final approval of the version to be published, and agreed to act as guarantor of the work (ensuring that questions related to any part of the work are appropriately investigated and resolved). All authors contributed to the article and approved the submitted version.

## Conflict of Interest

ZL, KL, UR, CJ, PL, and AA are current employees of the Pfizer and may hold stock options. The remaining authors declare that the research was conducted in the absence of any commercial or financial relationships that could be construed as a potential conflict of interest.

## Footnotes

1 http://pubmlst.org/cdifficile/

2 https://www.rcsb.org/

## References

[B1] Alonso C. D. Mahoney M. V. (2019). Bezlotoxumab for the prevention of *Clostridium difficile* infection: a review of current evidence and safety profile. *Infect. Drug Resist.* 12 1–9. 10.2147/idr.s159957 30588042 PMC6301304

[B2] Asempa T. E. Nicolau D. P. (2017). *Clostridium difficile* infection in the elderly: an update on management. *Clin. Interv. Aging* 12 1799–1809. 10.2147/cia.s149089 29123385 PMC5661493

[B3] Bartlett J. G. (2002). Clinical practice. Antibiotic-associated diarrhea. *N. Engl. J. Med.* 346 334–339. 11821511 10.1056/NEJMcp011603

[B4] Bezay N. Ayad A. Dubischar K. Firbas C. Hochreiter R. Kiermayr S. (2016). Safety, immunogenicity and dose response of VLA84, a new vaccine candidate against *Clostridium difficile*, in healthy volunteers. *Vaccine* 34 2585–2592. 10.1016/j.vaccine.2016.03.098 27079932

[B5] Bidet P. Barbut F. Lalande V. Burghoffer B. Petit J. C. (1999). Development of a new PCR-ribotyping method for *Clostridium difficile* based on ribosomal RNA gene sequencing. *FEMS Microbiol. Lett.* 175 261–266. 10.1111/j.1574-6968.1999.tb13629.x 10386377

[B6] Carter G. P. Chakravorty A. Pham Nguyen T. A. Mileto S. Schreiber F. Li L. (2015). Defining the Roles of TcdA and TcdB in localized gastrointestinal disease, systemic organ damage, and the host response during *clostridium difficile* infections. *mBio* 6:e00551. 10.1128/mBio.00551-15 PMC4453007 26037121

[B7] Chandrasekaran R. Lacy D. B. (2017). The role of toxins in *Clostridium difficile* infection. *FEMS Microbiol. Rev.* 41 723–750. 10.1093/femsre/fux048 29048477 PMC5812492

[B8] Cohen S. H. Gerding D. N. Johnson S. Kelly C. P. Loo V. G. Mcdonald L. C. (2010). Clinical practice guidelines for *Clostridium difficile* infection in adults: 2010 update by the society for healthcare epidemiology of America (SHEA) and the infectious diseases society of America (IDSA). *Infect. Control Hosp. Epidemiol.* 31 431–455. 10.1086/651706 20307191

[B9] den Dunnen J. T. Dalgleish R. Maglott D. R. Hart R. K. Greenblatt M. S. Mcgowan-Jordan J. (2016). HGVS Recommendations for the Description of Sequence Variants: 2016 Update. *Hum. Mutat.* 37 564–569. 10.1002/humu.22981 26931183

[B10] Dingle K. E. Griffiths D. Didelot X. Evans J. Vaughan A. Kachrimanidou M. (2011). Clinical *Clostridium difficile*: clonality and pathogenicity locus diversity. *PLoS One* 6:e19993. 10.1371/journal.pone.0019993 21625511 PMC3098275

[B11] Donald R. G. Flint M. Kalyan N. Johnson E. Witko S. E. Kotash C. (2013). A novel approach to generate a recombinant toxoid vaccine against *Clostridium difficile*. *Microbiology* 159 1254–1266. 10.1099/mic.0.066712-0 23629868 PMC3749728

[B12] Drudy D. Fanning S. Kyne L. (2007). Toxin A-negative, toxin B-positive *Clostridium difficile*. *Int. J. Infect. Dis.* 11 5–10. 10.1016/j.ijid.2006.04.003 16857405

[B13] Gerding D. N. Johnson S. Rupnik M. Aktories K. (2014). *Clostridium difficile* binary toxin CDT: mechanism, epidemiology, and potential clinical importance. *Gut Microbes* 5 15–27. 10.4161/gmic.26854 24253566 PMC4049931

[B14] Gerhard R. (2017). Receptors and Binding Structures for *Clostridium difficile* Toxins A and B. *Curr. Top. Microbiol. Immunol.* 406 79–96. 10.1007/82_2016_17 27380268

[B15] Gerhard R. Frenzel E. Goy S. Olling A. (2013). Cellular uptake of *Clostridium difficile* TcdA and truncated TcdA lacking the receptor binding domain. *J. Med. Microbiol.* 62 1414–1422. 10.1099/jmm.0.057828-0 23558138

[B16] Geric B. Johnson S. Gerding D. N. Grabnar M. Rupnik M. (2003). Frequency of binary toxin genes among *Clostridium difficile* strains that do not produce large clostridial toxins. *J. Clin. Microbiol.* 41 5227–5232. 10.1128/jcm.41.11.5227-5232.2003 14605169 PMC262504

[B17] Goorhuis A. Bakker D. Corver J. Debast S. B. Harmanus C. Notermans D. W. (2008). Emergence of *Clostridium difficile* infection due to a new hypervirulent strain, polymerase chain reaction ribotype 078. *Clin. Infect. Dis.* 47 1162–1170. 10.1086/592257 18808358

[B18] Griffiths D. Fawley W. Kachrimanidou M. Bowden R. Crook D. W. Fung R. (2010). Multilocus sequence typing of *Clostridium difficile*. *J. Clin. Microbiol.* 48 770–778. 20042623 10.1128/JCM.01796-09 PMC2832416

[B19] Huber C. A. Foster N. F. Riley T. V. Paterson D. L. (2013). Challenges for standardization of *Clostridium difficile* typing methods. *J. Clin. Microbiol.* 51 2810–2814. 10.1128/jcm.00143-13 23784128 PMC3754675

[B20] Imwattana K. Wangroongsarb P. Riley T. V. (2019). High prevalence and diversity of tcdA-negative and tcdB-positive, and non-toxigenic. *Clostridium difficile* in Thailand. *Anaerobe* 57 4–10. 10.1016/j.anaerobe.2019.03.008 30862468

[B21] Janezic S. Garneau J. R. Monot M. (2018). Comparative Genomics of *Clostridium difficile*. *Adv. Exp. Med. Biol.* 1050 59–75. 10.1007/978-3-319-72799-8_5 29383664

[B22] Janezic S. Rupnik M. (2019). Development and implementation of whole genome sequencing-based typing schemes for *Clostridioides difficile*. *Front. Public Health* 7:309. 10.3389/fpubh.2019.00309 31709221 PMC6821651

[B23] Kelly C. P. Pothoulakis C. Lamont J. T. (1994). *Clostridium difficile* colitis. *N. Engl. J. Med.* 330 257–262. 8043060 10.1056/NEJM199401273300406

[B24] Knetsch C. W. Lawley T. D. Hensgens M. P. Corver J. Wilcox M. W. Kuijper E. J. (2013). Current application and future perspectives of molecular typing methods to study *Clostridium difficile* infections. *Euro. Surveill.* 18:20381. 10.2807/ese.18.04.20381-en 23369393

[B25] Kuehne S. A. Collery M. M. Kelly M. L. Cartman S. T. Cockayne A. Minton N. P. (2014). Importance of toxin A, toxin B, and CDT in virulence of an epidemic *Clostridium difficile* strain. *J. Infect. Dis.* 209 83–86. 10.1093/infdis/jit426 23935202 PMC3864386

[B26] Kumar N. Browne H. P. Viciani E. Forster S. C. Clare S. Harcourt K. (2019). Adaptation of host transmission cycle during *Clostridium difficile* speciation. *Nat Genet.* 51 1–6. 31406348 10.1038/s41588-019-0478-8

[B27] LaFrance M. E. Farrow M. A. Chandrasekaran R. Sheng J. Rubin D. H. Lacy D. B. (2015). Identification of an epithelial cell receptor responsible for *Clostridium difficile* TcdB-induced cytotoxicity. *Proc. Natl. Acad. Sci. U.S.A.* 112 7073–7078. 10.1073/pnas.1500791112 26038560 PMC4460460

[B28] Lessa F. C. Mu Y. Bamberg W. M. Beldavs Z. G. Dumyati G. K. Dunn J. R. (2015). Burden of *Clostridium difficile* infection in the United States. *N. Engl. J. Med.* 372 825–834. 25714160 10.1056/NEJMoa1408913 PMC10966662

[B29] Liu X. S. Li W. G. Zhang W. Z. Wu Y. Lu J. X. (2018). Molecular Characterization of *Clostridium difficile* Isolates in China from 2010 to 2015. *Front Microbiol* 9:845. 10.3389/fmicb.2018.00845 29760687 PMC5936795

[B30] Loo V. G. Poirier L. Miller M. A. Oughton M. Libman M. D. Michaud S. (2005). A predominantly clonal multi-institutional outbreak of *Clostridium difficile*-associated diarrhea with high morbidity and mortality. *N. Engl. J. Med.* 353 2442–2449. 10.1056/nejmoa051639 16322602

[B31] Looney J. M. Edsall G. Ipsen J. Jr. Chasen W. H. (1956). Persistence of antitoxin levels after tetanus-toxoid inoculation in adults, and effect of a booster dose after various intervals. *N. Engl. J. Med.* 254 6–12. 10.1056/nejm195601052540102 13272863

[B32] Louie T. J. Miller M. A. Mullane K. M. Weiss K. Lentnek A. Golan Y. (2011). Fidaxomicin versus vancomycin for *Clostridium difficile* infection. *N. Engl. J. Med.* 364 422–431. 21288078 10.1056/NEJMoa0910812

[B33] McDonald L. C. Killgore G. E. Thompson A. Owens R. C. Jr. Kazakova S. V. (2005). An epidemic, toxin gene-variant strain of *Clostridium difficile*. *N. Engl. J. Med.* 353 2433–2441. 16322603 10.1056/NEJMoa051590

[B34] Monot M. Eckert C. Lemire A. Hamiot A. Dubois T. Tessier C. (2015). *Clostridium difficile*: new insights into the evolution of the pathogenicity locus. *Sci. Rep.* 5:15023. 10.1038/srep15023 PMC4597214 26446480

[B35] Orth P. Xiao L. Hernandez L. D. Reichert P. Sheth P. R. Beaumont M. (2014). Mechanism of action and epitopes of *Clostridium difficile* toxin B-neutralizing antibody bezlotoxumab revealed by X-ray crystallography. *J. Biol. Chem.* 289 18008–18021. 10.1074/jbc.m114.560748 24821719 PMC4140266

[B36] Reinert D. J. Jank T. Aktories K. Schulz G. E. (2005). Structural basis for the function of *Clostridium difficile* toxin B. *J. Mol. Biol.* 351 973–981. 10.1016/j.jmb.2005.06.071 16054646

[B37] Rupnik M. (2008). Heterogeneity of large clostridial toxins: importance of *Clostridium difficile* toxinotypes. *FEMS Microbiol. Rev.* 32 541–555. 10.1111/j.1574-6976.2008.00110.x 18397287

[B38] Rupnik M. Avesani V. Janc M. Von Eichel-Streiber C. Delmee M. (1998). A novel toxinotyping scheme and correlation of toxinotypes with serogroups of *Clostridium difficile* isolates. *J. Clin. Microbiol.* 36 2240–2247. 10.1128/jcm.36.8.2240-2247.1998 9665999 PMC105025

[B39] Rupnik M. Janezic S. (2016). An upate on *Clostridium difficile* toxinotyping. *J. Clin. Microbiol.* 54 13–18. 10.1128/jcm.02083-15 26511734 PMC4702747

[B40] Sanofi (2017). *Sanofi Ends Development of Clostridium difficile Vaccine.* Paris: Sanofi.

[B41] Sebaihia M. Wren B. W. Mullany P. Fairweather N. F. Minton N. Stabler R. (2006). The multidrug-resistant human pathogen *Clostridium difficile* has a highly mobile, mosaic genome. *Nat. Genet.* 38 779–786. 10.1038/ng1830 16804543

[B42] Secore S. Wang S. Doughtry J. Xie J. Miezeiewski M. Rustandi R. R. (2017). Development of a Novel Vaccine Containing Binary Toxin for the Prevention of *Clostridium difficile* disease with enhanced efficacy against NAP1 strains. *PLoS One* 12:e0170640. 10.1371/journal.pone.0170640 28125650 PMC5268477

[B43] Sheldon E. Kitchin N. Peng Y. Eiden J. Gruber W. Johnson E. (2016). A phase 1, placebo-controlled, randomized study of the safety, tolerability, and immunogenicity of a *Clostridium difficile* vaccine administered with or without aluminum hydroxide in healthy adults. *Vaccine* 34 2082–2091. 10.1016/j.vaccine.2016.03.010 26993331

[B44] Svenungsson B. Burman L. G. Jalakas-Pornull K. Lagergren A. Struwe J. Akerlund T. (2003). Epidemiology and molecular characterization of *Clostridium difficile* strains from patients with diarrhea: low disease incidence and evidence of limited cross-infection in a Swedish teaching hospital. *J. Clin. Microbiol.* 41 4031–4037. 10.1128/jcm.41.9.4031-4037.2003 12958221 PMC193849

[B45] Tao L. Zhang J. Meraner P. Tovaglieri A. Wu X. Gerhard R. (2016). Frizzled proteins are colonic epithelial receptors for *C. difficile* toxin B. *Nature* 538 350–355. 27680706 10.1038/nature19799 PMC5519134

[B46] Treangen T. J. Ondov B. D. Koren S. Phillippy A. M. (2014). The Harvest suite for rapid core-genome alignment and visualization of thousands of intraspecific microbial genomes. *Genome Biol.* 15: 524. 10.1186/s13059-014-0524-x PMC4262987 25410596

[B47] von Eichel-Streiber C. Zec-Pirnat I. Grabnar M. Rupnik M. (1999). A nonsense mutation abrogates production of a functional enterotoxin A in *Clostridium difficile* toxinotype VIII strains of serogroups F and X. *FEMS Microbiol. Lett.* 178 163–168. 10.1016/s0378-1097(99)00327-4 10483735

[B48] Voth D. E. Ballard J. D. (2005). *Clostridium difficile* toxins: mechanism of action and role in disease. *Clin. Microbiol. Rev.* 18 247–263. 10.1128/cmr.18.2.247-263.2005 15831824 PMC1082799

[B49] Yuan P. Zhang H. Cai C. Zhu S. Zhou Y. Yang X. (2015). Chondroitin sulfate proteoglycan 4 functions as the cellular receptor for *Clostridium difficile* toxin B. *Cell Res.* 25 157–168. 10.1038/cr.2014.169 25547119 PMC4650570

