# Supplementary figures and images for: Ribotype Classification of Clostridioides difficile Isolates Is Not Predictive of the Amino Acid Sequence Diversity of the Toxin Virulence Factors TcdA and TcdB
Source: Front Microbiol. 2020 Jun 19;11:1310. doi: 10.3389/fmicb.2020.01310 (PMC7318873; doi:10.3389/fmicb.2020.01310)

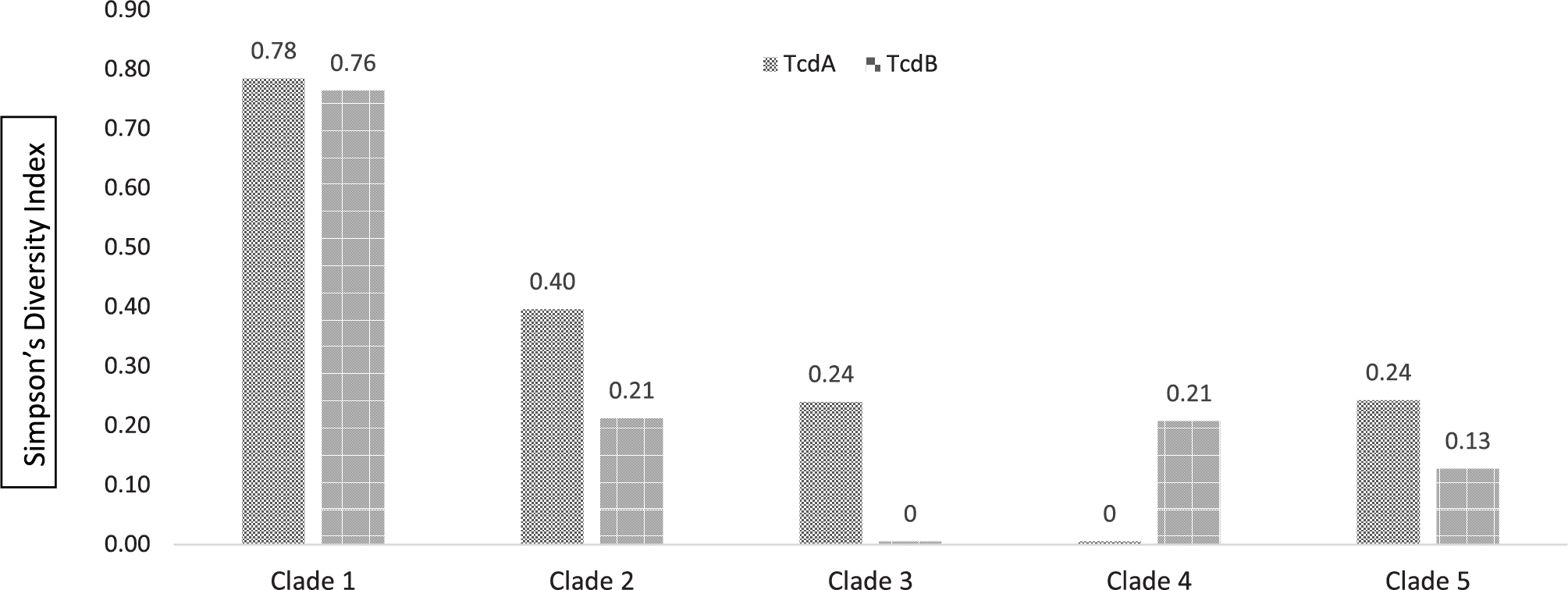

Supplement: Supplementary file 1 [file Image_1.JPEG]
