# Supplementary material for: Ribotype Classification of Clostridioides difficile Isolates Is Not Predictive of the Amino Acid Sequence Diversity of the Toxin Virulence Factors TcdA and TcdB
Source: Front Microbiol. 2020 Jun 19;11:1310. doi: 10.3389/fmicb.2020.01310 (PMC7318873; doi:10.3389/fmicb.2020.01310)
Supplement: Supplementary file 2 [file Table_1.docx]

Supplementary Table 1. *C difficile* isolates with atypical TcdA sequence characteristics.

| **Isolate ID** | **Clade** | **ST** | **Ribotype** | **TcdA Assignment** | **Descriptive Nomenclature  (den Dunnen et al., 2016)** | **TcdB Variant** |
| --- | --- | --- | --- | --- | --- | --- |
| PFECD0010 | 4 | 37 | 017 | truncated | p.Q47* | TcdB003 |
| PFECD0009 | 4 | 37 | 017 | truncated | p.Q47* | TcdB003 |
| PFECD0083 | 4 | 37 | 017 | truncated | p.Q47* | TcdB003 |
| PFECD0086 | 4 | 37 | 017 | truncated | p.Q47* | TcdB003 |
| PFECD0090 | 4 | 37 | 017 | truncated | p.Q47* | TcdB003 |
| PFECD0093 | 4 | 37 | 017 | truncated | p.Q47* | TcdB003 |
| PFECD0099 | 4 | 37 | 017 | truncated | p.Q47* | TcdB003 |
| PFECD0243 | 4 | 37 | 017 | truncated | p.Q47* | TcdB003 |
| PFECD0269 | 4 | 37 | 017 | truncated | p.Q47* | TcdB003 |
| PFECD0275 | 4 | 37 | 017 | truncated | p.Q47* | TcdB003 |
| PFECD0342 | 4 | 37 | 017 | truncated | p.Q47* | TcdB003 |
| PFECD0349 | 4 | 37 | 017 | truncated | p.Q47* | TcdB003 |
| PFECD0391 | 4 | 37 | 017 | truncated | p.Q47* | TcdB003 |
| PFECD0396 | 4 | 37 | 017 | truncated | p.Q47* | TcdB003 |
| PFECD0292 | 2 | 1 | 176 | truncated | p.V57* | TcdB002 |
| PFECD0300 | 4 | 37 | 017 | truncated | p.D108* | TcdB003 |
| PFECD0106 | 5 | 11 | 045 | truncated | p.P196* | TcdB004 |
| PFECD0140 | 2 | 62 | 591 | truncated | p.G699* | TcdB032 |
| PFECD0187 | 2 | 567 | 095 | truncated | p.G699* | TcdB038 |
| PFECD0192 | 2 | 567 | 095 | truncated | p.G699* | TcdB038 |
| PFECD0186 | 2 | 1 | 027 | TcdA050 | p.Val2163_Leu2195del | TcdB002 |
| PFECD0234 | 5 | 11 | 413 | TcdA051 | p.Val2211_Phe2385del | TcdB004 |
| PFECD0523 | 2 | 1 | 027 | TcdA052 | p.Y2172* | TcdB002 |
| PFECD0111 | 5 | 11 | 045 | TcdA053 | p.Y2172* | TcdB004 |
| PFECD0203 | 1 | 3 | 023 | TcdA054 | p.G2327* | TcdB041 |

‘Supplementary Figure 1. The Simpson’s Diversity Index (SDI) of TcdA and TcdB.

The *C. difficile* strains in this study were grouped to one of five clades according to their MLST genotype. The SDI of TcdA and TcdB variants expressed by the strains within the respective clades are plotted. The SDI is used as a quantitative measure of the diversity and distribution of toxin variants within each clade. The higher the index, the greater the number and distribution of toxin variants among strains grouped to that clade. Likewise, the lower the index, the less diverse/restricted the toxin variants are within the clade. With one exception, the SDI of TcdA variants was greater than that of TcdB variants in each clade.
