# Supplementary material for: Ribotype Classification of Clostridioides difficile Isolates Is Not Predictive of the Amino Acid Sequence Diversity of the Toxin Virulence Factors TcdA and TcdB
Source: Front Microbiol. 2020 Jun 19;11:1310. doi: 10.3389/fmicb.2020.01310 (PMC7318873; doi:10.3389/fmicb.2020.01310)
Supplement: Supplementary file 3 [file Table_2.doc]

Supplementary Table 2. Molecular characteristics for the 478 *C. difficile* isolates that are listed in this study.

| **Strain ID** | **Ribotype** | **ST (MLST)** | **Clade** | **TcdA variant** | **TcdB variant** | **Binary toxin** |
| --- | --- | --- | --- | --- | --- | --- |
| PFECD0001 | 001 | 3 | 1 | TcdA010 | TcdB012 | NEGATIVE |
| PFECD0002 | 001 | 3 | 1 | TcdA010 | TcdB012 | NEGATIVE |
| PFECD0003 | 018/356 | 122 | 1 | TcdA017 | TcdB016 | POSITIVE |
| PFECD0004 | 002 | 8 | 1 | TcdA010 | TcdB012 | NEGATIVE |
| PFECD0005 | 027 | 12 | 1 | TcdA018 | TcdB010 | NEGATIVE |
| PFECD0006 | 014/020 | 2 | 1 | TcdA010 | TcdB012 | NEGATIVE |
| PFECD0007 | 015 | 10 | 1 | TcdA014 | TcdB005 | NEGATIVE |
| PFECD0008 | 015 | 10 | 1 | TcdA014 | TcdB005 | NEGATIVE |
| PFECD0009 | 017 | 37 | 4 | truncated | TcdB003 | NEGATIVE |
| PFECD0010 | 017 | 37 | 4 | truncated | TcdB003 | NEGATIVE |
| PFECD0011 | 014/020 | 13 | 1 | TcdA003 | TcdB007 | NEGATIVE |
| PFECD0012 | 023 | 5 | 3 | TcdA019 | TcdB006 | POSITIVE |
| PFECD0013 | 002 | 8 | 1 | TcdA010 | TcdB012 | NEGATIVE |
| PFECD0014 | 027 | 1 | 2 | TcdA007 | TcdB002 | POSITIVE |
| PFECD0015 | 027 | 1 | 2 | TcdA007 | TcdB002 | POSITIVE |
| PFECD0016 | 023 | 5 | 3 | TcdA019 | TcdB006 | POSITIVE |
| PFECD0017 | 050 | 35 | 1 | TcdA004 | TcdB008 | NEGATIVE |
| PFECD0018 | 053 | 63 | 1 | TcdA012 | TcdB008 | NEGATIVE |
| PFECD0019 | 053 | 63 | 1 | TcdA012 | TcdB008 | NEGATIVE |
| PFECD0020 | 053 | 63 | 1 | TcdA012 | TcdB008 | NEGATIVE |
| PFECD0021 | 056 | 34 | 1 | TcdA011 | TcdB015 | NEGATIVE |
| PFECD0022 | 018/356 | 122 | 1 | TcdA017 | TcdB016 | POSITIVE |
| PFECD0023 | 057 | 55 | 1 | TcdA015 | TcdB012 | NEGATIVE |
| PFECD0024 | 095 | 2 | 1 | TcdA010 | TcdB012 | NEGATIVE |
| PFECD0025 | 095 | 49 | 1 | TcdA010 | TcdB012 | NEGATIVE |
| PFECD0026 | 014/020 | 2 | 1 | TcdA010 | TcdB012 | NEGATIVE |
| PFECD0027 | 078/126 | 11 | 5 | TcdA013 | TcdB004 | POSITIVE |
| PFECD0028 | 078/126 | 11 | 5 | TcdA013 | TcdB004 | POSITIVE |
| PFECD0029 | 078/126 | 11 | 5 | TcdA013 | TcdB004 | POSITIVE |
| PFECD0030 | 078/126 | 11 | 5 | TcdA013 | TcdB004 | POSITIVE |
| PFECD0031 | 081 | 9 | 1 | TcdA009 | TcdB013 | NEGATIVE |
| PFECD0032 | 087 | uncharacterized | not defined | TcdA009 | TcdB001 | NEGATIVE |
| PFECD0033 | 095 | 2 | 1 | TcdA010 | TcdB012 | NEGATIVE |
| PFECD0034 | 078/126 | 11 | 5 | TcdA013 | TcdB004 | POSITIVE |
| PFECD0035 | 106 | 42 | 1 | TcdA002 | TcdB009 | NEGATIVE |
| PFECD0036 | 014/020 | 54 | 1 | TcdA005 | TcdB001 | NEGATIVE |
| PFECD0037 | 018/356 | 122 | 1 | TcdA017 | TcdB016 | POSITIVE |
| PFECD0038 | 078/126 | 11 | 5 | TcdA013 | TcdB004 | POSITIVE |
| PFECD0039 | 019 | 41 | 2 | TcdA016 | TcdB011 | POSITIVE |
| PFECD0040 | 018/356 | 122 | 1 | TcdA017 | TcdB016 | POSITIVE |
| PFECD0041 | 014/020 | 102 | 1 | TcdA010 | TcdB014 | NEGATIVE |
| PFECD0042 | 027 | 1 | 2 | TcdA007 | TcdB002 | POSITIVE |
| PFECD0043 | 056 | 53 | 1 | TcdA008 | TcdB008 | NEGATIVE |
| PFECD0044 | 053 | 63 | 1 | TcdA012 | TcdB008 | NEGATIVE |
| PFECD0045 | 019 | 41 | 2 | TcdA016 | TcdB011 | POSITIVE |
| PFECD0046 | 014/020 | 54 | 1 | TcdA005 | TcdB001 | NEGATIVE |
| PFECD0047 | 003 | 12 | 1 | TcdA018 | TcdB010 | POSITIVE |
| PFECD0048 | 019 | 67 | 2 | TcdA016 | TcdB011 | POSITIVE |
| PFECD0049 | 001 | 3 | 1 | TcdA010 | TcdB012 | NEGATIVE |
| PFECD0078 | 012 | 54 | 1 | TcdA001 | TcdB001 | NEGATIVE |
| PFECD0080 | 151 (CE) | 200 | not defined | deletion | TcdB029 | NEGATIVE |
| PFECD0081 | 126 | 11 | 5 | TcdA013 | TcdB004 | POSITIVE |
| PFECD0082 | 126 | 11 | 5 | TcdA013 | TcdB004 | POSITIVE |
| PFECD0083 | 017 | 37 | 4 | truncated | TcdB003 | NEGATIVE |
| PFECD0084 | 023 | 5 | 3 | TcdA019 | TcdB006 | POSITIVE |
| PFECD0085 | 078 | 11 | 5 | TcdA013 | TcdB004 | POSITIVE |
| PFECD0086 | 017 | 37 | 4 | truncated | TcdB003 | NEGATIVE |
| PFECD0087 | 023 | 5 | 3 | TcdA019 | TcdB006 | POSITIVE |
| PFECD0088 | SLO 176 | 245 | 1 | TcdA009 | TcdB018 | NEGATIVE |
| PFECD0090 | 017 | 37 | 4 | truncated | TcdB003 | NEGATIVE |
| PFECD0091 | 027 | 1 | 2 | TcdA007 | TcdB002 | POSITIVE |
| PFECD0092 | 023 | 5 | 3 | TcdA019 | TcdB006 | POSITIVE |
| PFECD0093 | 017 | 37 | 4 | truncated | TcdB003 | NEGATIVE |
| PFECD0094 | 023 | 5 | 3 | TcdA019 | TcdB006 | POSITIVE |
| PFECD0095 | SLO 079 | 114 | 2 | TcdA020 | TcdB019 | POSITIVE |
| PFECD0096 | 078 | 11 | 5 | TcdA013 | TcdB004 | POSITIVE |
| PFECD0097 | 027 | 1 | 2 | TcdA007 | TcdB002 | POSITIVE |
| PFECD0098 | 023 | 5 | 3 | TcdA019 | TcdB006 | POSITIVE |
| PFECD0099 | 017 | 37 | 4 | truncated | TcdB003 | NEGATIVE |
| PFECD0100 | 023 | 5 | 3 | TcdA019 | TcdB006 | POSITIVE |
| PFECD0101 | 023 | 5 | 3 | TcdA019 | TcdB006 | POSITIVE |
| PFECD0102 | SLO 176 | 245 | 1 | TcdA009 | TcdB018 | NEGATIVE |
| PFECD0103 | SLO 126 | 562 | 2 | TcdA021 | TcdB020 | NEGATIVE |
| PFECD0104 | 027 | 1 | 2 | TcdA007 | TcdB002 | POSITIVE |
| PFECD0105 | 027 | 1 | 2 | TcdA007 | TcdB002 | POSITIVE |
| PFECD0106 | 045 | 11 | 5 | truncated | TcdB004 | POSITIVE |
| PFECD0107 | 027 | 1 | 2 | TcdA007 | TcdB002 | POSITIVE |
| PFECD0108 | SLO 079 | 114 | 2 | TcdA020 | TcdB019 | POSITIVE |
| PFECD0110 | 034 | 223 | 2 | TcdA026 | TcdB002 | POSITIVE |
| PFECD0111 | 045 | 11 | 5 | TcdA053 | TcdB004 | POSITIVE |
| PFECD0112 | 255 | 34 | 1 | TcdA011 | TcdB015 | NEGATIVE |
| PFECD0113 | 126 | 11 | 5 | TcdA013 | TcdB004 | POSITIVE |
| PFECD0114 | 126 | 11 | 5 | TcdA013 | TcdB004 | POSITIVE |
| PFECD0115 | 126 | 11 | 5 | TcdA013 | TcdB004 | POSITIVE |
| PFECD0116 | 258 | 58 | 1 | TcdA011 | TcdB015 | NEGATIVE |
| PFECD0117 | 078 | 11 | 5 | TcdA013 | TcdB004 | POSITIVE |
| PFECD0118 | 126 | 11 | 5 | TcdA013 | TcdB004 | POSITIVE |
| PFECD0119 | 255 | 34 | 1 | TcdA011 | TcdB015 | NEGATIVE |
| PFECD0120 | SLO 247 | 565 | 2 | TcdA027 | TcdB002 | POSITIVE |
| PFECD0122 | 255 | 34 | 1 | TcdA011 | TcdB015 | NEGATIVE |
| PFECD0123 | SLO 176 | 245 | 1 | TcdA009 | TcdB018 | NEGATIVE |
| PFECD0124 | 078 | 11 | 5 | TcdA013 | TcdB004 | POSITIVE |
| PFECD0125 | 258 | 58 | 1 | TcdA010 | TcdB026 | NEGATIVE |
| PFECD0126 | SLO 176 | 245 | 1 | TcdA009 | TcdB018 | NEGATIVE |
| PFECD0127 | 078 | 11 | 5 | TcdA013 | TcdB004 | POSITIVE |
| PFECD0128 | 255 | 34 | 1 | TcdA011 | TcdB015 | NEGATIVE |
| PFECD0129 | 176 (CE) | 1 | 2 | TcdA007 | TcdB002 | POSITIVE |
| PFECD0130 | SLO 128 | 41 | 2 | TcdA029 | TcdB027 | POSITIVE |
| PFECD0131 | SLO 176 | 245 | 1 | TcdA009 | TcdB018 | NEGATIVE |
| PFECD0132 | 078 | 11 | 5 | TcdA013 | TcdB004 | POSITIVE |
| PFECD0133 | 126 | 11 | 5 | TcdA013 | TcdB004 | POSITIVE |
| PFECD0134 | SLO 079 | 114 | 2 | TcdA020 | TcdB019 | POSITIVE |
| PFECD0135 | 023 | 5 | 3 | TcdA019 | TcdB006 | POSITIVE |
| PFECD0136 | 126 | 11 | 5 | TcdA013 | TcdB004 | POSITIVE |
| PFECD0137 | SLO 098 | 167 | 5 | deletion | TcdB030 | POSITIVE |
| PFECD0138 | SLO 101 | 166 | 5 | deletion | TcdB035 | POSITIVE |
| PFECD0140 | 591(CE) | 62 | 2 | truncated | TcdB032 | POSITIVE |
| PFECD0141 | SLO 228 | 226 | 2 | TcdA049 | TcdB033 | POSITIVE |
| PFECD0143 | 019 | 67 | 2 | TcdA016 | TcdB011 | POSITIVE |
| PFECD0144 | 056 | 36 | 1 | TcdA003 | TcdB012 | NEGATIVE |
| PFECD0145 | 027 | 1 | 2 | TcdA007 | TcdB002 | POSITIVE |
| PFECD0146 | 002 | 8 | 1 | TcdA010 | TcdB012 | NEGATIVE |
| PFECD0147 | 106 | 42 | 1 | TcdA002 | TcdB009 | NEGATIVE |
| PFECD0148 | 106 | 42 | 1 | TcdA002 | TcdB009 | NEGATIVE |
| PFECD0149 | 027 | 1 | 2 | TcdA007 | TcdB002 | POSITIVE |
| PFECD0150 | 106 | 42 | 1 | TcdA002 | TcdB009 | NEGATIVE |
| PFECD0151 | 056 | 34 | 1 | TcdA011 | TcdB015 | NEGATIVE |
| PFECD0152 | 002 | 8 | 1 | TcdA010 | TcdB012 | NEGATIVE |
| PFECD0153 | 001 | 3 | 1 | TcdA010 | TcdB012 | NEGATIVE |
| PFECD0154 | 027 | 1 | 2 | TcdA007 | TcdB002 | POSITIVE |
| PFECD0155 | 106 | 42 | 1 | TcdA002 | TcdB009 | NEGATIVE |
| PFECD0156 | 001 | 2 | 1 | TcdA010 | TcdB012 | NEGATIVE |
| PFECD0157 | 027 | 1 | 2 | TcdA007 | TcdB002 | POSITIVE |
| PFECD0158 | 027 | 1 | 2 | TcdA007 | TcdB002 | POSITIVE |
| PFECD0159 | 002 | 8 | 1 | TcdA010 | TcdB012 | NEGATIVE |
| PFECD0160 | 012 | 54 | 1 | TcdA001 | TcdB001 | NEGATIVE |
| PFECD0161 | 014/020 | 110 | 1 | TcdA010 | TcdB012 | NEGATIVE |
| PFECD0162 | 013 | 44 | 1 | TcdA009 | TcdB013 | NEGATIVE |
| PFECD0163 | 014/020 | 14 | 1 | TcdA010 | TcdB012 | NEGATIVE |
| PFECD0164 | 027 | 1 | 2 | TcdA007 | TcdB002 | POSITIVE |
| PFECD0167 | 027 | 1 | 2 | TcdA007 | TcdB002 | NEGATIVE |
| PFECD0168 | 050 | 16 | 1 | TcdA014 | TcdB021 | NEGATIVE |
| PFECD0169 | 001 | 21 | 1 | TcdA015 | TcdB036 | NEGATIVE |
| PFECD0171 | 027 | 1 | 2 | TcdA007 | TcdB002 | POSITIVE |
| PFECD0173 | 027 | 1 | 2 | TcdA007 | TcdB002 | POSITIVE |
| PFECD0174 | 056 | 53 | 1 | TcdA008 | TcdB008 | NEGATIVE |
| PFECD0175 | 033 | 566 | 1 | TcdA009 | TcdB013 | NEGATIVE |
| PFECD0176 | 014/020 | 2 | 1 | TcdA010 | TcdB012 | NEGATIVE |
| PFECD0177 | 001 | 3 | 1 | TcdA010 | TcdB012 | NEGATIVE |
| PFECD0178 | 005 | 6 | 1 | TcdA010 | TcdB012 | NEGATIVE |
| PFECD0179 | 027 | 1 | 2 | TcdA007 | TcdB002 | POSITIVE |
| PFECD0180 | 056 | 34 | 1 | TcdA011 | TcdB015 | NEGATIVE |
| PFECD0181 | 014/020 | 26 | 1 | deletion | deletion | NEGATIVE |
| PFECD0182 | 027 | 1 | 2 | TcdA007 | TcdB002 | POSITIVE |
| PFECD0183 | 106 | 42 | 1 | TcdA002 | TcdB009 | NEGATIVE |
| PFECD0185 | 027 | 1 | 2 | TcdA007 | TcdB002 | POSITIVE |
| PFECD0186 | 027 | 1 | 2 | TcdA050 | TcdB002 | POSITIVE |
| PFECD0187 | 095 | 567 | 2 | truncated | TcdB038 | POSITIVE |
| PFECD0189 | 056 | 49 | 1 | TcdA010 | TcdB012 | NEGATIVE |
| PFECD0190 | 027 | 1 | 2 | TcdA007 | TcdB002 | POSITIVE |
| PFECD0191 | 106 | 42 | 1 | TcdA002 | TcdB009 | NEGATIVE |
| PFECD0192 | 095 | 567 | 2 | truncated | TcdB038 | POSITIVE |
| PFECD0193 | 027 | 1 | 2 | TcdA007 | TcdB002 | POSITIVE |
| PFECD0194 | 027 | 1 | 2 | TcdA007 | TcdB002 | POSITIVE |
| PFECD0195 | 027 | 1 | 2 | TcdA007 | TcdB002 | POSITIVE |
| PFECD0196 | 015 | 44 | 1 | TcdA014 | TcdB021 | NEGATIVE |
| PFECD0197 | 027 | 1 | 2 | TcdA007 | TcdB002 | POSITIVE |
| PFECD0199 | 027 | 1 | 2 | TcdA007 | TcdB002 | POSITIVE |
| PFECD0200 | 002 | 8 | 1 | TcdA010 | TcdB012 | NEGATIVE |
| PFECD0201 | 056 | 34 | 1 | TcdA011 | TcdB015 | NEGATIVE |
| PFECD0202 | 014/020 | 49 | 1 | TcdA010 | TcdB012 | NEGATIVE |
| PFECD0203 | 023 | 3 | 1 | TcdA054 | TcdB041 | NEGATIVE |
| PFECD0204 | 027/198 | 1 | 2 | TcdA007 | TcdB002 | POSITIVE |
| PFECD0205 | 027 | 1 | 2 | TcdA007 | TcdB002 | POSITIVE |
| PFECD0206 | 039 | 26 | 1 | deletion | deletion | NEGATIVE |
| PFECD0207 | UNK-7 | 172 | 1 | TcdA010 | TcdB001 | NEGATIVE |
| PFECD0208 | 001 | 3 | 1 | TcdA010 | TcdB012 | NEGATIVE |
| PFECD0209 | 001 | 3 | 1 | TcdA010 | TcdB012 | NEGATIVE |
| PFECD0210 | 013 | 35 | 1 | TcdA025 | TcdB008 | NEGATIVE |
| PFECD0211 | 002 | 8 | 1 | TcdA010 | TcdB012 | NEGATIVE |
| PFECD0213 | 010 | 15 | 1 | deletion | deletion | NEGATIVE |
| PFECD0214 | 029 | 16 | 1 | TcdA014 | TcdB021 | NEGATIVE |
| PFECD0215 | 003 | 12 | 1 | TcdA018 | TcdB010 | NEGATIVE |
| PFECD0216 | UNK-7 | 13 | 1 | TcdA003 | TcdB007 | NEGATIVE |
| PFECD0217 | 056 | 34 | 1 | TcdA011 | TcdB015 | NEGATIVE |
| PFECD0218 | 056 | 34 | 1 | TcdA011 | TcdB015 | NEGATIVE |
| PFECD0219 | 087 | 563 | 1 | deletion | deletion | NEGATIVE |
| PFECD0220 | 033 | 5 | 3 | TcdA019 | TcdB006 | POSITIVE |
| PFECD0221 | 027 | 1 | 2 | TcdA007 | TcdB002 | POSITIVE |
| PFECD0222 | 003 | 75 | 1 | TcdA022 | truncated | NEGATIVE |
| PFECD0223 | 014/020 | 2 | 1 | TcdA010 | TcdB012 | NEGATIVE |
| PFECD0225 | 027 | 1 | 2 | TcdA007 | TcdB002 | POSITIVE |
| PFECD0226 | 013 | 44 | 1 | TcdA014 | TcdB021 | NEGATIVE |
| PFECD0227 | 176 | 1 | 2 | TcdA007 | TcdB002 | POSITIVE |
| PFECD0228 | 070 | 55 | 1 | TcdA015 | TcdB012 | NEGATIVE |
| PFECD0229 | 003 | 12 | 1 | TcdA018 | truncated | NEGATIVE |
| PFECD0230 | 001 | 3 | 1 | TcdA010 | TcdB012 | NEGATIVE |
| PFECD0231 | 056 | 34 | 1 | TcdA011 | TcdB015 | NEGATIVE |
| PFECD0232 | 026 | 6 | 1 | TcdA010 | TcdB012 | NEGATIVE |
| PFECD0233 | 018/356 | 42 | 1 | TcdA002 | TcdB009 | NEGATIVE |
| PFECD0234 | 413 | 11 | 5 | TcdA051 | TcdB004 | POSITIVE |
| PFECD0235 | 010 | 15 | 1 | deletion | deletion | NEGATIVE |
| PFECD0236 | 039 | 26 | 1 | deletion | deletion | NEGATIVE |
| PFECD0237 | 014/020 | 2 | 1 | TcdA010 | TcdB012 | NEGATIVE |
| PFECD0238 | 078 | 11 | 5 | TcdA013 | TcdB004 | POSITIVE |
| PFECD0239 | 070 | 564 | 1 | TcdA015 | TcdB012 | NEGATIVE |
| PFECD0241 | 027 | 1 | 2 | TcdA023 | TcdB002 | POSITIVE |
| PFECD0242 | 010 | 15 | 1 | deletion | deletion | NEGATIVE |
| PFECD0243 | 017 | 37 | 4 | truncated | TcdB003 | NEGATIVE |
| PFECD0244 | 106 | 6 | 1 | TcdA010 | TcdB023 | NEGATIVE |
| PFECD0245 | 002 | 8 | 1 | TcdA010 | TcdB012 | NEGATIVE |
| PFECD0246 | 027 | 239 | 1 | TcdA002 | TcdB008 | NEGATIVE |
| PFECD0247 | 106 | 3 | 1 | TcdA024 | TcdB024 | NEGATIVE |
| PFECD0248 | 027 | 239 | 1 | TcdA002 | TcdB008 | NEGATIVE |
| PFECD0249 | 001 | 3 | 1 | TcdA010 | TcdB012 | NEGATIVE |
| PFECD0250 | 001 | 3 | 1 | TcdA010 | TcdB012 | NEGATIVE |
| PFECD0251 | 027 | 1 | 2 | TcdA007 | TcdB002 | POSITIVE |
| PFECD0253 | 027 | 1 | 2 | TcdA007 | TcdB002 | POSITIVE |
| PFECD0254 | 014/020 | 49 | 1 | TcdA010 | TcdB012 | NEGATIVE |
| PFECD0255 | 029 | 16 | 1 | TcdA014 | TcdB021 | NEGATIVE |
| PFECD0256 | 014/020 | 14 | 1 | TcdA010 | TcdB012 | NEGATIVE |
| PFECD0257 | 014/020 | 2 | 1 | TcdA010 | TcdB012 | NEGATIVE |
| PFECD0258 | 014/020 | 13 | 1 | TcdA003 | TcdB007 | NEGATIVE |
| PFECD0259 | 014/020 | 8 | 1 | TcdA010 | TcdB012 | NEGATIVE |
| PFECD0260 | 027 | 1 | 2 | TcdA007 | TcdB002 | POSITIVE |
| PFECD0261 | 014/020 | 14 | 1 | TcdA010 | TcdB012 | NEGATIVE |
| PFECD0262 | 078 | 11 | 5 | TcdA013 | TcdB004 | POSITIVE |
| PFECD0263 | 014/020 | 14 | 1 | TcdA010 | TcdB012 | NEGATIVE |
| PFECD0264 | 001 | 3 | 1 | TcdA010 | TcdB012 | NEGATIVE |
| PFECD0265 | 014/020 | 2 | 1 | TcdA010 | TcdB028 | NEGATIVE |
| PFECD0266 | 010 | 15 | 1 | deletion | deletion | NEGATIVE |
| PFECD0267 | 027 | 1 | 2 | TcdA007 | TcdB002 | POSITIVE |
| PFECD0268 | 070 | 55 | 1 | TcdA015 | TcdB012 | NEGATIVE |
| PFECD0269 | 017 | 37 | 4 | truncated | TcdB003 | NEGATIVE |
| PFECD0270 | 027 | 1 | 2 | TcdA007 | TcdB002 | POSITIVE |
| PFECD0271 | 018/356 | 42 | 1 | TcdA002 | TcdB009 | NEGATIVE |
| PFECD0272 | 001 | 3 | 1 | TcdA010 | TcdB012 | NEGATIVE |
| PFECD0274 | 027 | 1 | 2 | TcdA007 | TcdB002 | POSITIVE |
| PFECD0275 | 017 | 37 | 4 | truncated | TcdB003 | NEGATIVE |
| PFECD0276 | 027 | 1 | 2 | TcdA007 | TcdB002 | POSITIVE |
| PFECD0277 | 001 | 3 | 1 | TcdA010 | TcdB012 | NEGATIVE |
| PFECD0278 | 027 | 1 | 2 | TcdA007 | TcdB002 | POSITIVE |
| PFECD0279 | 198 | 1 | 2 | TcdA007 | TcdB002 | POSITIVE |
| PFECD0280 | 027 | 1 | 2 | TcdA007 | TcdB002 | POSITIVE |
| PFECD0281 | 027 | 1 | 2 | TcdA007 | TcdB002 | POSITIVE |
| PFECD0282 | 002 | 8 | 1 | TcdA010 | TcdB012 | NEGATIVE |
| PFECD0283 | 053 | 4 | 1 | TcdA039 | TcdB001 | NEGATIVE |
| PFECD0284 | 001 | 3 | 1 | TcdA010 | TcdB012 | NEGATIVE |
| PFECD0285 | 054 | 7 | 1 | deletion | deletion | NEGATIVE |
| PFECD0286 | 027 | 1 | 2 | TcdA007 | TcdB002 | POSITIVE |
| PFECD0287 | 001 | 3 | 1 | TcdA010 | TcdB012 | NEGATIVE |
| PFECD0288 | 081 | 1 | 2 | TcdA007 | TcdB002 | POSITIVE |
| PFECD0289 | 014/020 | 110 | 1 | TcdA010 | TcdB012 | NEGATIVE |
| PFECD0290 | 176 | 1 | 2 | TcdA007 | TcdB002 | POSITIVE |
| PFECD0291 | 013 | 571 | 1 | TcdA010 | TcdB012 | NEGATIVE |
| PFECD0292 | 176 | 1 | 2 | truncated | TcdB002 | POSITIVE |
| PFECD0293 | 014/020 | 2 | 1 | TcdA010 | TcdB012 | NEGATIVE |
| PFECD0294 | 400 | 185 | 1 | TcdA044 | TcdB008 | NEGATIVE |
| PFECD0296 | 002 | 8 | 1 | TcdA010 | TcdB012 | NEGATIVE |
| PFECD0297 | 001 | 48 | 1 | deletion | deletion | NEGATIVE |
| PFECD0298 | 053 | 29 | 1 | deletion | deletion | NEGATIVE |
| PFECD0299 | 056 | 45 | 1 | TcdA014 | TcdB037 | NEGATIVE |
| PFECD0300 | 017 | 37 | 4 | truncated | TcdB003 | NEGATIVE |
| PFECD0301 | 176 | 29 | 1 | deletion | deletion | NEGATIVE |
| PFECD0302 | 176 | 1 | 2 | TcdA007 | TcdB002 | POSITIVE |
| PFECD0303 | 003 | 12 | 1 | TcdA018 | TcdB010 | NEGATIVE |
| PFECD0304 | 176 | 1 | 2 | TcdA007 | TcdB002 | POSITIVE |
| PFECD0306 | 400 | 185 | 1 | TcdA044 | TcdB008 | NEGATIVE |
| PFECD0307 | 078 | 11 | 5 | TcdA013 | TcdB004 | POSITIVE |
| PFECD0308 | 053 | 54 | 1 | TcdA001 | TcdB001 | NEGATIVE |
| PFECD0309 | 078 | 11 | 5 | TcdA013 | TcdB004 | POSITIVE |
| PFECD0310 | 078/126 | 11 | 5 | TcdA013 | TcdB004 | POSITIVE |
| PFECD0311 | 207 | 110 | 1 | TcdA010 | TcdB012 | NEGATIVE |
| PFECD0312 | 011 | 29 | 1 | deletion | deletion | NEGATIVE |
| PFECD0313 | 010 | 15 | 1 | deletion | deletion | NEGATIVE |
| PFECD0314 | 014/020 | 13 | 1 | TcdA003 | TcdB007 | NEGATIVE |
| PFECD0316 | 026 | 6 | 1 | TcdA010 | TcdB012 | NEGATIVE |
| PFECD0317 | 027 | 1 | 2 | TcdA007 | TcdB002 | POSITIVE |
| PFECD0318 | 001 | 3 | 1 | TcdA010 | TcdB012 | NEGATIVE |
| PFECD0319 | 095 | 3 | 1 | TcdA010 | TcdB012 | NEGATIVE |
| PFECD0320 | 001 | 3 | 1 | TcdA010 | TcdB012 | NEGATIVE |
| PFECD0321 | 001 | 12 | 1 | TcdA018 | TcdB001 | NEGATIVE |
| PFECD0322 | 027 | 1 | 2 | TcdA007 | TcdB002 | POSITIVE |
| PFECD0323 | 012 | 29 | 1 | deletion | deletion | NEGATIVE |
| PFECD0324 | 003 | 12 | 1 | TcdA018 | TcdB001 | NEGATIVE |
| PFECD0325 | 010 | 15 | 1 | deletion | deletion | NEGATIVE |
| PFECD0326 | 027 | 1 | 2 | TcdA007 | TcdB002 | POSITIVE |
| PFECD0327 | 027 | 1 | 2 | TcdA007 | TcdB002 | POSITIVE |
| PFECD0328 | 013 | 44 | 1 | TcdA014 | TcdB021 | NEGATIVE |
| PFECD0330 | 027 | 1 | 2 | TcdA007 | TcdB002 | POSITIVE |
| PFECD0331 | 027 | 1 | 2 | TcdA007 | TcdB002 | POSITIVE |
| PFECD0332 | 027 | 1 | 2 | TcdA007 | TcdB002 | POSITIVE |
| PFECD0333 | 027 | 1 | 2 | TcdA007 | TcdB002 | POSITIVE |
| PFECD0334 | 001 | 3 | 1 | TcdA010 | TcdB012 | NEGATIVE |
| PFECD0335 | 027 | 1 | 2 | TcdA007 | TcdB002 | POSITIVE |
| PFECD0336 | 027 | 1 | 2 | TcdA007 | TcdB002 | POSITIVE |
| PFECD0337 | 027 | 1 | 2 | TcdA007 | TcdB002 | POSITIVE |
| PFECD0338 | 045 | 161 | 5 | TcdA037 | truncated | POSITIVE |
| PFECD0339 | 027 | 1 | 2 | TcdA007 | TcdB002 | POSITIVE |
| PFECD0340 | 198 | 1 | 2 | TcdA007 | TcdB002 | POSITIVE |
| PFECD0341 | 026 | 6 | 1 | TcdA010 | TcdB012 | NEGATIVE |
| PFECD0342 | 017 | 37 | 4 | truncated | TcdB003 | NEGATIVE |
| PFECD0343 | 176 | 1 | 2 | TcdA007 | TcdB002 | POSITIVE |
| PFECD0344 | 014/020 | 49 | 1 | TcdA010 | TcdB012 | NEGATIVE |
| PFECD0345 | 176 | 1 | 2 | TcdA007 | TcdB002 | POSITIVE |
| PFECD0346 | 078 | 11 | 5 | TcdA013 | TcdB004 | POSITIVE |
| PFECD0347 | 039 | 26 | 1 | deletion | deletion | NEGATIVE |
| PFECD0348 | 126 | 11 | 5 | TcdA013 | TcdB004 | POSITIVE |
| PFECD0349 | 017 | 37 | 4 | truncated | TcdB003 | NEGATIVE |
| PFECD0350 | 001 | 298 | 4 | deletion | deletion | NEGATIVE |
| PFECD0351 | 176 | 1 | 2 | TcdA007 | TcdB002 | POSITIVE |
| PFECD0352 | 014/020 | 14 | 1 | TcdA010 | TcdB012 | NEGATIVE |
| PFECD0353 | 103 | 221 | 3 | TcdA045 | TcdB006 | POSITIVE |
| PFECD0354 | 126 | 11 | 5 | TcdA013 | TcdB004 | POSITIVE |
| PFECD0355 | 050 | 35 | 1 | TcdA025 | TcdB008 | NEGATIVE |
| PFECD0356 | 029 | 16 | 1 | TcdA014 | TcdB021 | NEGATIVE |
| PFECD0357 | 014/020 | 2 | 1 | TcdA010 | TcdB012 | NEGATIVE |
| PFECD0358 | 070 | 55 | 1 | TcdA015 | TcdB012 | NEGATIVE |
| PFECD0359 | 001 | 3 | 1 | TcdA010 | TcdB012 | NEGATIVE |
| PFECD0360 | 018/356 | 42 | 1 | TcdA002 | TcdB009 | NEGATIVE |
| PFECD0361 | 018/356 | 42 | 1 | TcdA002 | TcdB009 | NEGATIVE |
| PFECD0362 | 078/126 | 11 | 5 | TcdA013 | TcdB004 | POSITIVE |
| PFECD0363 | 078/126 | 11 | 5 | TcdA013 | TcdB004 | POSITIVE |
| PFECD0364 | 001 | 3 | 1 | TcdA010 | TcdB012 | NEGATIVE |
| PFECD0365 | 078/126 | 11 | 5 | TcdA013 | TcdB004 | POSITIVE |
| PFECD0366 | 026 | 19 | 1 | TcdA040 | TcdB008 | NEGATIVE |
| PFECD0367 | 018/356 | 42 | 1 | TcdA002 | TcdB009 | NEGATIVE |
| PFECD0369 | 027 | 239 | 1 | TcdA002 | TcdB008 | NEGATIVE |
| PFECD0370 | 013 | 44 | 1 | TcdA014 | TcdB021 | NEGATIVE |
| PFECD0371 | 207 | 110 | 1 | TcdA010 | TcdB012 | NEGATIVE |
| PFECD0372 | 070 | 55 | 1 | TcdA015 | TcdB012 | NEGATIVE |
| PFECD0373 | 014/020 | 2 | 1 | TcdA010 | TcdB012 | NEGATIVE |
| PFECD0374 | 027 | 1 | 2 | TcdA007 | TcdB002 | POSITIVE |
| PFECD0375 | 198/027 | 1 | 2 | TcdA007 | TcdB002 | POSITIVE |
| PFECD0376 | 106 | 42 | 1 | TcdA002 | TcdB009 | NEGATIVE |
| PFECD0377 | 018/356 | 42 | 1 | TcdA002 | TcdB009 | NEGATIVE |
| PFECD0378 | 001 | 3 | 1 | TcdA010 | TcdB012 | NEGATIVE |
| PFECD0379 | 056 | 34 | 1 | TcdA011 | TcdB015 | NEGATIVE |
| PFECD0380 | 078/126 | 11 | 5 | TcdA013 | TcdB004 | POSITIVE |
| PFECD0381 | 027 | 1 | 2 | TcdA007 | TcdB002 | POSITIVE |
| PFECD0382 | 014/020 | 2 | 1 | TcdA010 | TcdB012 | NEGATIVE |
| PFECD0383 | 106 | 42 | 1 | TcdA002 | TcdB009 | NEGATIVE |
| PFECD0384 | 078/126 | 11 | 5 | TcdA013 | TcdB004 | POSITIVE |
| PFECD0385 | 033 | 22 | 3 | TcdA019 | TcdB006 | POSITIVE |
| PFECD0386 | 078/126 | 11 | 5 | TcdA013 | TcdB004 | POSITIVE |
| PFECD0387 | 002 | 8 | 1 | TcdA010 | TcdB012 | NEGATIVE |
| PFECD0389 | 012 | 29 | 1 | deletion | deletion | NEGATIVE |
| PFECD0390 | 097 | 21 | 1 | TcdA015 | TcdB036 | NEGATIVE |
| PFECD0391 | 017 | 37 | 4 | truncated | TcdB003 | NEGATIVE |
| PFECD0392 | 050 | 10 | 1 | TcdA014 | TcdB021 | NEGATIVE |
| PFECD0393 | 106 | 42 | 1 | TcdA002 | TcdB009 | NEGATIVE |
| PFECD0394 | 078/126 | 11 | 5 | TcdA013 | TcdB004 | POSITIVE |
| PFECD0395 | 014/020 | 49 | 1 | TcdA010 | TcdB012 | NEGATIVE |
| PFECD0396 | 017 | 37 | 4 | truncated | TcdB003 | NEGATIVE |
| PFECD0397 | 001 | 3 | 1 | TcdA010 | TcdB012 | NEGATIVE |
| PFECD0398 | 027 | 1 | 2 | TcdA007 | TcdB002 | POSITIVE |
| PFECD0399 | 027 | 1 | 2 | TcdA007 | TcdB002 | POSITIVE |
| PFECD0400 | 001 | 3 | 1 | TcdA010 | TcdB012 | NEGATIVE |
| PFECD0401 | 078/126 | 564 | 1 | TcdA015 | TcdB012 | NEGATIVE |
| PFECD0402 | 081 | 9 | 1 | TcdA009 | TcdB013 | NEGATIVE |
| PFECD0403 | 026 | 19 | 1 | TcdA040 | TcdB008 | NEGATIVE |
| PFECD0404 | 010 | 15 | 1 | deletion | deletion | NEGATIVE |
| PFECD0405 | 001 | 3 | 1 | TcdA010 | TcdB012 | NEGATIVE |
| PFECD0406 | 081 | 1 | 2 | TcdA007 | TcdB002 | POSITIVE |
| PFECD0408 | 027 | 1 | 2 | TcdA007 | TcdB002 | POSITIVE |
| PFECD0409 | 207 | 102 | 1 | TcdA010 | TcdB014 | NEGATIVE |
| PFECD0410 | 001 | 3 | 1 | TcdA010 | TcdB012 | NEGATIVE |
| PFECD0411 | 027 | 1 | 2 | TcdA007 | TcdB002 | POSITIVE |
| PFECD0412 | 054 | 43 | 1 | TcdA041 | TcdB012 | NEGATIVE |
| PFECD0413 | 056 | 58 | 1 | TcdA042 | TcdB015 | NEGATIVE |
| PFECD0414 | 014/020 | 2 | 1 | TcdA010 | TcdB012 | NEGATIVE |
| PFECD0415 | 078/126 | 512 | 1 | TcdA015 | TcdB012 | NEGATIVE |
| PFECD0416 | 001 | 3 | 1 | TcdA010 | TcdB012 | NEGATIVE |
| PFECD0417 | 014/020 | 2 | 1 | TcdA010 | TcdB012 | NEGATIVE |
| PFECD0418 | 106 | 42 | 1 | TcdA002 | TcdB009 | NEGATIVE |
| PFECD0419 | 056 | 36 | 1 | TcdA003 | TcdB012 | NEGATIVE |
| PFECD0420 | 002 | 8 | 1 | TcdA010 | TcdB012 | NEGATIVE |
| PFECD0421 | 153 | 268 | 1 | TcdA002 | TcdB008 | NEGATIVE |
| PFECD0422 | 011 | 33 | 1 | TcdA043 | TcdB039 | NEGATIVE |
| PFECD0423 | 056 | 36 | 1 | TcdA003 | TcdB012 | NEGATIVE |
| PFECD0424 | 027 | 1 | 2 | TcdA007 | TcdB002 | POSITIVE |
| PFECD0425 | 002 | 8 | 1 | TcdA010 | TcdB012 | NEGATIVE |
| PFECD0426 | 002 | 8 | 1 | TcdA010 | TcdB012 | NEGATIVE |
| PFECD0427 | 002 | 8 | 1 | TcdA010 | TcdB012 | NEGATIVE |
| PFECD0428 | 027 | 1 | 2 | TcdA007 | TcdB002 | POSITIVE |
| PFECD0429 | 001 | 3 | 1 | TcdA010 | TcdB012 | NEGATIVE |
| PFECD0430 | 027 | 1 | 2 | TcdA007 | TcdB002 | POSITIVE |
| PFECD0431 | 027 | 1 | 2 | TcdA007 | TcdB002 | POSITIVE |
| PFECD0432 | 001 | 3 | 1 | TcdA010 | TcdB012 | NEGATIVE |
| PFECD0433 | 001 | 3 | 1 | TcdA010 | TcdB012 | NEGATIVE |
| PFECD0434 | 002 | 8 | 1 | TcdA010 | TcdB012 | NEGATIVE |
| PFECD0435 | 027 | 1 | 2 | TcdA007 | TcdB002 | POSITIVE |
| PFECD0436 | 027 | 1 | 2 | TcdA007 | TcdB002 | POSITIVE |
| PFECD0437 | 018/356 | 17 | 1 | TcdA010 | TcdB012 | NEGATIVE |
| PFECD0438 | 014/020 | 2 | 1 | TcdA010 | TcdB012 | NEGATIVE |
| PFECD0439 | 010 | 15 | 1 | deletion | deletion | NEGATIVE |
| PFECD0440 | 010 | 15 | 1 | deletion | deletion | NEGATIVE |
| PFECD0441 | 078/126 | 11 | 5 | TcdA013 | TcdB004 | POSITIVE |
| PFECD0442 | 027 | 1 | 2 | TcdA007 | TcdB002 | POSITIVE |
| PFECD0444 | 027 | 1 | 2 | TcdA007 | TcdB002 | POSITIVE |
| PFECD0445 | 027 | 1 | 2 | TcdA007 | TcdB002 | POSITIVE |
| PFECD0446 | 027 | 1 | 2 | TcdA007 | TcdB002 | POSITIVE |
| PFECD0447 | 009 | 3 | 1 | deletion | deletion | NEGATIVE |
| PFECD0448 | 002 | 8 | 1 | TcdA010 | TcdB012 | NEGATIVE |
| PFECD0449 | 050 | 44 | 1 | TcdA014 | TcdB021 | NEGATIVE |
| PFECD0450 | 050 | 35 | 1 | TcdA004 | TcdB008 | NEGATIVE |
| PFECD0451 | 014/020 | 2 | 1 | TcdA010 | TcdB012 | NEGATIVE |
| PFECD0452 | 018/356 | 42 | 1 | TcdA002 | TcdB009 | NEGATIVE |
| PFECD0454 | 078/126 | 568 | 3 | TcdA046 | TcdB006 | POSITIVE |
| PFECD0455 | 002 | 8 | 1 | TcdA010 | TcdB012 | NEGATIVE |
| PFECD0456 | 002 | 8 | 1 | TcdA010 | TcdB012 | NEGATIVE |
| PFECD0457 | 026 | 6 | 1 | TcdA010 | TcdB012 | NEGATIVE |
| PFECD0458 | 027 | 1 | 2 | TcdA007 | TcdB002 | POSITIVE |
| PFECD0459 | 027 | 1 | 2 | TcdA007 | TcdB002 | POSITIVE |
| PFECD0460 | 056 | 34 | 1 | TcdA011 | TcdB015 | NEGATIVE |
| PFECD0461 | 050 | 35 | 1 | TcdA025 | TcdB008 | NEGATIVE |
| PFECD0462 | 014/020 | 2 | 1 | TcdA010 | TcdB012 | NEGATIVE |
| PFECD0463 | 014/020 | 2 | 1 | TcdA010 | TcdB012 | NEGATIVE |
| PFECD0464 | 026 | 6 | 1 | TcdA010 | TcdB012 | NEGATIVE |
| PFECD0465 | 056 | 36 | 1 | TcdA003 | TcdB012 | NEGATIVE |
| PFECD0466 | 027 | 1 | 2 | TcdA048 | TcdB002 | POSITIVE |
| PFECD0467 | 039 | 569 | 4 | deletion | deletion | NEGATIVE |
| PFECD0468 | 027 | 1 | 2 | TcdA007 | TcdB002 | POSITIVE |
| PFECD0469 | 001 | 3 | 1 | TcdA010 | TcdB012 | NEGATIVE |
| PFECD0470 | 014/020 | 2 | 1 | TcdA015 | TcdB012 | NEGATIVE |
| PFECD0472 | 001 | 3 | 1 | TcdA010 | TcdB012 | NEGATIVE |
| PFECD0473 | 002 | 8 | 1 | TcdA010 | TcdB012 | NEGATIVE |
| PFECD0474 | 027 | 1 | 2 | TcdA007 | TcdB002 | POSITIVE |
| PFECD0475 | 027 | 1 | 2 | TcdA007 | TcdB002 | POSITIVE |
| PFECD0476 | 027 | 1 | 2 | TcdA048 | TcdB002 | POSITIVE |
| PFECD0477 | 014/020 | 2 | 1 | TcdA010 | TcdB008 | NEGATIVE |
| PFECD0478 | 027 | 1 | 2 | TcdA007 | TcdB002 | POSITIVE |
| PFECD0479 | 207 | 110 | 1 | TcdA010 | TcdB012 | NEGATIVE |
| PFECD0480 | 001 | 3 | 1 | TcdA010 | TcdB012 | NEGATIVE |
| PFECD0481 | 027 | 1 | 2 | TcdA048 | TcdB002 | POSITIVE |
| PFECD0482 | 027 | 1 | 2 | TcdA007 | TcdB002 | POSITIVE |
| PFECD0483 | 027 | 1 | 2 | TcdA048 | TcdB002 | POSITIVE |
| PFECD0484 | 027 | 1 | 2 | TcdA048 | TcdB002 | POSITIVE |
| PFECD0485 | 027 | 1 | 2 | TcdA048 | TcdB002 | POSITIVE |
| PFECD0486 | 251 | 67 | 2 | TcdA047 | TcdB043 | POSITIVE |
| PFECD0487 | 078/126 | 11 | 5 | TcdA013 | TcdB004 | POSITIVE |
| PFECD0488 | 010 | 26 | 1 | deletion | deletion | NEGATIVE |
| PFECD0489 | 027 | 1 | 2 | TcdA048 | TcdB002 | POSITIVE |
| PFECD0490 | 014/020 | 2 | 1 | TcdA010 | TcdB012 | NEGATIVE |
| PFECD0491 | 027 | 1 | 2 | TcdA048 | TcdB002 | POSITIVE |
| PFECD0492 | 014/020 | 2 | 1 | TcdA010 | TcdB008 | NEGATIVE |
| PFECD0493 | 010 | 15 | 1 | deletion | deletion | NEGATIVE |
| PFECD0494 | 011 | 36 | 1 | TcdA003 | TcdB012 | NEGATIVE |
| PFECD0495 | 010 | 15 | 1 | deletion | deletion | NEGATIVE |
| PFECD0496 | 078/126 | 11 | 5 | TcdA013 | TcdB004 | POSITIVE |
| PFECD0497 | 001 | 3 | 1 | TcdA010 | TcdB012 | NEGATIVE |
| PFECD0498 | 014/020 | 13 | 1 | TcdA010 | TcdB012 | NEGATIVE |
| PFECD0499 | 023 | 5 | 3 | TcdA019 | TcdB006 | POSITIVE |
| PFECD0500 | 014/020 | 14 | 1 | TcdA010 | TcdB012 | NEGATIVE |
| PFECD0501 | 053 | 63 | 1 | TcdA012 | TcdB008 | NEGATIVE |
| PFECD0502 | 001 | 3 | 1 | TcdA010 | TcdB012 | NEGATIVE |
| PFECD0503 | 003 | 12 | 1 | TcdA018 | TcdB010 | NEGATIVE |
| PFECD0504 | 039 | 26 | 1 | deletion | deletion | NEGATIVE |
| PFECD0505 | 014/020 | 2 | 1 | TcdA010 | TcdB012 | NEGATIVE |
| PFECD0506 | 078/126 | 11 | 5 | TcdA013 | TcdB004 | POSITIVE |
| PFECD0507 | 018/356 | 7 | 1 | TcdA010 | TcdB012 | NEGATIVE |
| PFECD0508 | 027 | 1 | 2 | TcdA007 | TcdB002 | POSITIVE |
| PFECD0509 | 014/020 | 2 | 1 | TcdA010 | TcdB012 | NEGATIVE |
| PFECD0510 | 097 | 21 | 1 | TcdA015 | TcdB036 | NEGATIVE |
| PFECD0511 | 010 | 15 | 1 | deletion | deletion | NEGATIVE |
| PFECD0512 | 027 | 1 | 2 | TcdA007 | TcdB002 | POSITIVE |
| PFECD0513 | 014/020 | 2 | 1 | TcdA010 | TcdB012 | NEGATIVE |
| PFECD0514 | 014/020 | 103 | 1 | TcdA010 | TcdB008 | NEGATIVE |
| PFECD0515 | 106 | 42 | 1 | TcdA002 | TcdB042 | NEGATIVE |
| PFECD0516 | 027 | 1 | 2 | TcdA007 | TcdB002 | POSITIVE |
| PFECD0517 | 056 | 58 | 1 | TcdA010 | TcdB026 | NEGATIVE |
| PFECD0518 | 106 | 42 | 1 | TcdA002 | TcdB009 | NEGATIVE |
| PFECD0519 | 012 | 54 | 1 | TcdA001 | TcdB001 | NEGATIVE |
| PFECD0520 | 027 | 1 | 2 | TcdA007 | TcdB002 | POSITIVE |
| PFECD0521 | 014/020 | 14 | 1 | TcdA010 | TcdB012 | NEGATIVE |
| PFECD0522 | 056 | 34 | 1 | TcdA011 | TcdB015 | NEGATIVE |
| PFECD0523 | 027 | 1 | 2 | TcdA052 | TcdB002 | POSITIVE |
| PFECD0524 | 027 | 1 | 2 | TcdA007 | TcdB002 | POSITIVE |
| PFECD0525 | 106 | 42 | 1 | TcdA002 | TcdB009 | NEGATIVE |
| PFECD0526 | 106 | 42 | 1 | TcdA002 | TcdB009 | NEGATIVE |
| PFECD0527 | 010 | 15 | 1 | deletion | deletion | NEGATIVE |
| PFECD0528 | 007 | 100 | 1 | deletion | deletion | NEGATIVE |
| PFECD0529 | 106 | 42 | 1 | TcdA002 | TcdB009 | NEGATIVE |
| PFECD0530 | 054 | 43 | 1 | TcdA041 | TcdB012 | NEGATIVE |
| PFECD0532 | 027 | 1 | 2 | TcdA007 | TcdB002 | POSITIVE |
| PFECD0533 | 056 | 34 | 1 | TcdA011 | TcdB015 | NEGATIVE |
| PFECD0535 | 001 | 10 | 1 | TcdA014 | TcdB021 | NEGATIVE |
| PFECD0536 | 002 | 8 | 1 | TcdA010 | TcdB012 | NEGATIVE |
